# Mitochondrial DNA is critical for longevity and metabolism of transmission stage *Trypanosoma brucei*

**DOI:** 10.1371/journal.ppat.1007195

**Published:** 2018-07-18

**Authors:** Caroline E. Dewar, Paula MacGregor, Sinclair Cooper, Matthew K. Gould, Keith R. Matthews, Nicholas J. Savill, Achim Schnaufer

**Affiliations:** Centre for Immunity, Infection and Evolution, Institute of Immunology & Infection Research, University of Edinburgh, Edinburgh, United Kingdom; University Wuerzburg, GERMANY

## Abstract

The sleeping sickness parasite *Trypanosoma brucei* has a complex life cycle, alternating between a mammalian host and the tsetse fly vector. A tightly controlled developmental programme ensures parasite transmission between hosts as well as survival within them and involves strict regulation of mitochondrial activities. In the glucose-rich bloodstream, the replicative ‘slender’ stage is thought to produce ATP exclusively via glycolysis and uses the mitochondrial F_1_F_O_-ATP synthase as an ATP hydrolysis-driven proton pump to generate the mitochondrial membrane potential (ΔΨm). The ‘procyclic’ stage in the glucose-poor tsetse midgut depends on mitochondrial catabolism of amino acids for energy production, which involves oxidative phosphorylation with ATP production via the F_1_F_O_-ATP synthase. Both modes of the F_1_F_O_ enzyme critically depend on F_O_ subunit *a*, which is encoded in the parasite’s mitochondrial DNA (kinetoplast or kDNA). Comparatively little is known about mitochondrial function and the role of kDNA in non-replicative ‘stumpy’ bloodstream forms, a developmental stage essential for disease transmission. Here we show that the L262P mutation in the nuclear-encoded F_1_ subunit γ that permits survival of ‘slender’ bloodstream forms lacking kDNA (‘akinetoplastic’ forms), via F_O_-independent generation of ΔΨm, also permits their differentiation into stumpy forms. However, these akinetoplastic stumpy cells lack a ΔΨm and have a reduced lifespan *in vitro* and in mice, which significantly alters the within-host dynamics of the parasite. We further show that generation of ΔΨm in stumpy parasites and their ability to use α-ketoglutarate to sustain viability depend on F_1_-ATPase activity. Surprisingly, however, loss of ΔΨm does not reduce stumpy life span. We conclude that the L262P γ subunit mutation does not enable F_O_-independent generation of ΔΨm in stumpy cells, most likely as a consequence of mitochondrial ATP production in these cells. In addition, kDNA-encoded genes other than F_O_ subunit *a* are important for stumpy form viability.

## Author summary

African trypanosomes are single cellular eukaryotes transmitted by tsetse flies that cause important diseases in humans and their livestock. For survival these parasites depend on their mitochondrion, an organelle that has its own genome (‘mtDNA’) and that is traditionally viewed as the ‘power plant’ of cells, but that has other essential roles as well. Interfering with mtDNA is an important part of how some anti-trypanosomatid drugs work. Most mitochondrial research in trypanosomes in the past has focused on forms of the sleeping sickness parasite that proliferate in the mammalian bloodstream or in the insect midgut, as these can be readily cultured in the laboratory. As a consequence, relatively little is known about mitochondrial biology of the so-called ‘stumpy’ form, a non-replicative stage that is critical for transmission to tsetse flies. In this study we use a mouse infection model to show that a certain gene mutation permits formation of stumpy forms that lack mtDNA. However, without mtDNA these parasites have an altered mitochondrial metabolism and a reduced life span, which tells us that stumpy forms depend on additional mtDNA-encoded genes that are not required by the proliferative bloodstream form.

## Introduction

The parasitic protist *Trypanosoma brucei* undergoes a complex life cycle involving stages within the mammalian bloodstream and its tsetse fly vector. In the bloodstream of the mammalian host, the cell population exhibits two major morphotypes: the proliferative long slender bloodstream form (BSF) and the cell cycle-arrested stumpy form. Differentiation from the slender BSF to the stumpy form is triggered upon high slender parasite numbers [1]. The emergence of cell cycle-arrested stumpy forms prevents parasitaemia increasing further, prolonging host survival, and results in the characteristic waves of parasitaemia seen in bloodstream infections in rodents. This density dependent differentiation has been shown to be induced by a stumpy induction factor (SIF) via a form of quorum sensing [2].

The stumpy form is insect-transmissible and is preadapted to survive within the low glucose environment of the tsetse fly midgut, where it differentiates to the procyclic (PCF) tsetse midgut form of the parasite. PCF are able to generate ATP using mitochondrial energy production pathways, involving both oxidative and substrate-level phosphorylation [3–6]. In contrast, ATP production in the slender BSF is thought to solely involve non-mitochondrial glycolysis, utilising the glucose-rich environment found within the mammalian bloodstream [7,8]. Comparatively little is known about the metabolic requirements of stumpy forms, but studies have demonstrated an increase in the abundance of many mitochondrial proteins in the stumpy life cycle form compared to the slender BSF, including subunits of the mitochondrial respiratory complexes and key mitochondrial metabolic enzyme activities such as pyruvate dehydrogenase, α-ketoglutarate (α-KG) dehydrogenase, acetate:succinate CoA-transferase (ASCT) and succinyl-CoA synthetase (SCoAS) [9–12]. Accordingly, stumpy forms can utilise both glucose and α-KG as carbon sources for mitochondrial substrate level phosphorylation, at least *in vitro* [9,10,13]. Cytochromes have not been detected in stumpy forms [10], but the presence of an abbreviated oxidative phosphorylation pathway consisting of respiratory complexes I (cI; NADH:ubiquinone oxidoreductase) and V (F_1_F_O_-ATP synthase) and the trypanosome alternative oxidase (AOX) has been proposed [14].

The single mitochondrion of *T*. *brucei* contains a complex genome, termed kinetoplast DNA or kDNA and comprising of maxicircles and minicircles [15]. The maxicircle corresponds to the mitochondrial DNA of other organisms and encodes subunits of the respiratory chain and mitoribosome. Messenger RNAs for 12 of the 18 protein-coding genes require RNA editing by uridylyl insertion and deletion for maturation, a post-transcriptional process directed by guide RNAs, which are encoded in the minicircles. The parasite’s kDNA is essential in the long slender BSF and PCF stages of the life cycle [16–19]. In the latter, this is presumably due to a requirement for kDNA-encoded subunits of respiratory complexes III (cytochrome *bc*_*1*_ complex; subunit *b* encoded in kDNA), IV (cytochrome oxidase; subunits I, II and III encoded in kDNA) and the F_1_F_O_-ATP synthase (subunit *a* encoded in kDNA). These complexes are key constituents of the oxidative phosphorylation pathway that is required to generate ATP in that stage of the life cycle [4,6]. In the slender BSF, the F_1_F_O_-ATP synthase operates as an ATP hydrolysis-driven proton pump to maintain the essential electrical potential across the inner mitochondrial membrane (ΔΨm) [20–24]. As a consequence, the F_1_F_O_-ATP synthase, along with its kDNA-encoded subunit *a*, is essential in long slender BSF *T*. *brucei*. It is not known which, if any, mitochondrial genes are essential in the stumpy form.

*T*. *b*. *evansi* and *T*. *b*. *equiperdum* are naturally occurring ‘dyskinetoplastic’ subspecies of *T*. *brucei* that survive in the complete (akinetoplastidy; kDNA^0^) or partial (kDNA^-^) absence of kDNA [25,26] (it should be noted that the taxonomic status of these organisms is controversial, see [27,28]). Interestingly, *T*. *b*. *evansi* and *T*. *b*. *equiperdum* are generally considered to be “monomorphic”: they remain in the slender form within the host, and, respectively, are transmitted mechanically via the mouthparts of hematophagous flies or sexually in horses. Stumpy forms have only been reported occasionally in field samples of kDNA^-^ and kDNA^0^ strains [29–31]. However no surviving laboratory-grown strains of kDNA^0^ trypanosomes show any ability for pleomorphism [18,32]. It is not known whether the lack of kDNA influences the monomorphism displayed by these kDNA^0^ parasites, or whether the loss of pleomorphism and kDNA are independently selected. This question is potentially relevant for the spread of drug resistance, as kDNA independence is associated with reduced susceptibility to anti-trypanosomatid compounds belonging to the phenanthridines and diamidines [33].

We decided to investigate kDNA function and mitochondrial metabolism in stumpy forms by utilising a mutation in the nuclear-encoded γ subunit of the F_1_F_O_-ATPase, L262Pγ, that allows kDNA-independence in slender BSF *T*. *b*. *brucei* [16]. We generated pleomorphic slender BSF *T*. *b*. *brucei* cell lines that express L262Pγ, allowing the deletion of kDNA by acriflavine to produce clonal kDNA^0^ cells, and studied the within-host dynamics of these cell lines in mice and their physiology *in vitro*. Mouse infections showed that kDNA^0^ *T*. *b*. *brucei* are in fact capable of differentiating to the stumpy form *in vivo*. However, we found that kDNA^0^ stumpy cells have a shortened lifespan *in vivo* and *in vitro*. These kDNA^0^ stumpy cells are unable to sustain viability with α-KG as carbon source and do not have a Δψm. Treatment of kDNA^+^ stumpy form with the F_1_F_O_-ATPase inhibitor azide abolished Δψm. Our findings suggest that, like in slender BSF, the F_1_F_O_-ATP synthase generates the Δψm in early stumpy forms by acting as a proton-pumping ATPase. However, in contrast to slender BSF, the L262Pγ mutation is not sufficient to maintain full viability of kDNA^0^ stumpy forms. Our study provides new insight into stumpy form energy metabolism and kDNA function.

## Results

### Pleomorphic cells independent of kDNA are able to differentiate to stumpy forms *in vivo*

In order to be able to investigate the requirement for functional kDNA in the differentiation of *T*. *brucei* from slender to stumpy form, we replaced one allele of the nuclear-encoded F_1_F_O_-ATPase subunit γ with a version with the L262P mutation (L262Pγ) in the pleomorphic cell line EATRO 1125 (AnTat1.1 90:13) [34] (generating cell line WT/L262Pγ; see Table 1 for a list of cell lines used in this study). This mutation fully compensates for the requirement for kDNA in slender bloodstream form *T*. *brucei* [16]. We introduced a wild type version (WTγ) into the same parental cell line to generate an otherwise isogenic control (WT/WTγ). We generated two cell lines lacking kDNA (kDNA^0^ #1 and #2) from two distinct clones of genotype WT/L262Pγ (#1 and #2) by treatment with acriflavine [16]; we obtained a third WT/L262Pγ (kDNA^0^) cell line (#3) fortuitously after spontaneous loss of kDNA. We confirmed absence of kDNA in all three cell lines by PCR and microscopically (S1A and S1B Fig). *In vitro*, WT/L262Pγ kDNA^+^ and kDNA^0^ cell lines grew at the same rate as the WT/WTγ cell line, showing that both modifications had no effect on the viability of the cells under these conditions (S1C Fig). As expected, cells expressing an L262Pγ allele, regardless of the presence of kDNA, were resistant to 10 nM EtBr [33], unlike cells expressing solely WTγ, which died within 4–5 days of treatment (S1C Fig).

**Table 1:** Cell lines used in this study.

Cell line	Derived from:	Genotype	Description
WT/WTγ	EATRO 1125 AnTat 1.1 90:13	*ATPγ/Δatpγ*::*atpγWT+PURO*	One F_1_F_O_-ATP synthase subunit γ allele replaced with a WT copy and the puromycin resistance gene (PURO)
WT/L262Pγ #1	EATRO 1125 AnTat 1.1 90:13	*ATPγ/Δatpγ*::*atpγL262P+PURO*	One F_1_F_O_-ATP synthase subunit γ allele replaced with a copy with the L262P mutation and the puromycin resistance gene (PURO)
WT/L262Pγ #2	EATRO 1125 AnTat 1.1 90:13	*ATPγ/Δatpγ*::*atpγL262P+PURO*	One F_1_F_O_-ATP synthase subunit γ allele replaced with a copy with the L262P mutation and the puromycin resistance gene (PURO)
WT/L262Pγ (kDNA^0^) #1	WT/L262Pγ #1	*ATPγ/Δatpγ*::*atpγL262P+PURO*	Cell line WT/L262Pγ #1 induced to lose kDNA through exposure to acriflavine
WT/L262Pγ (kDNA^0^) #2	WT/L262Pγ #2	*ATPγ/Δatpγ*::*atpγL262P+PURO*	Cell line WT/L262Pγ #2 induced to lose kDNA through exposure to acriflavine
WT/L262Pγ (kDNA^0^) #3	WT/L262Pγ #3	*ATPγ/Δatpγ*::*atpγL262P+BSD*	Cell line WT/L262Pγ #3 after spontaneous loss of kDNA

**Fig 1:**
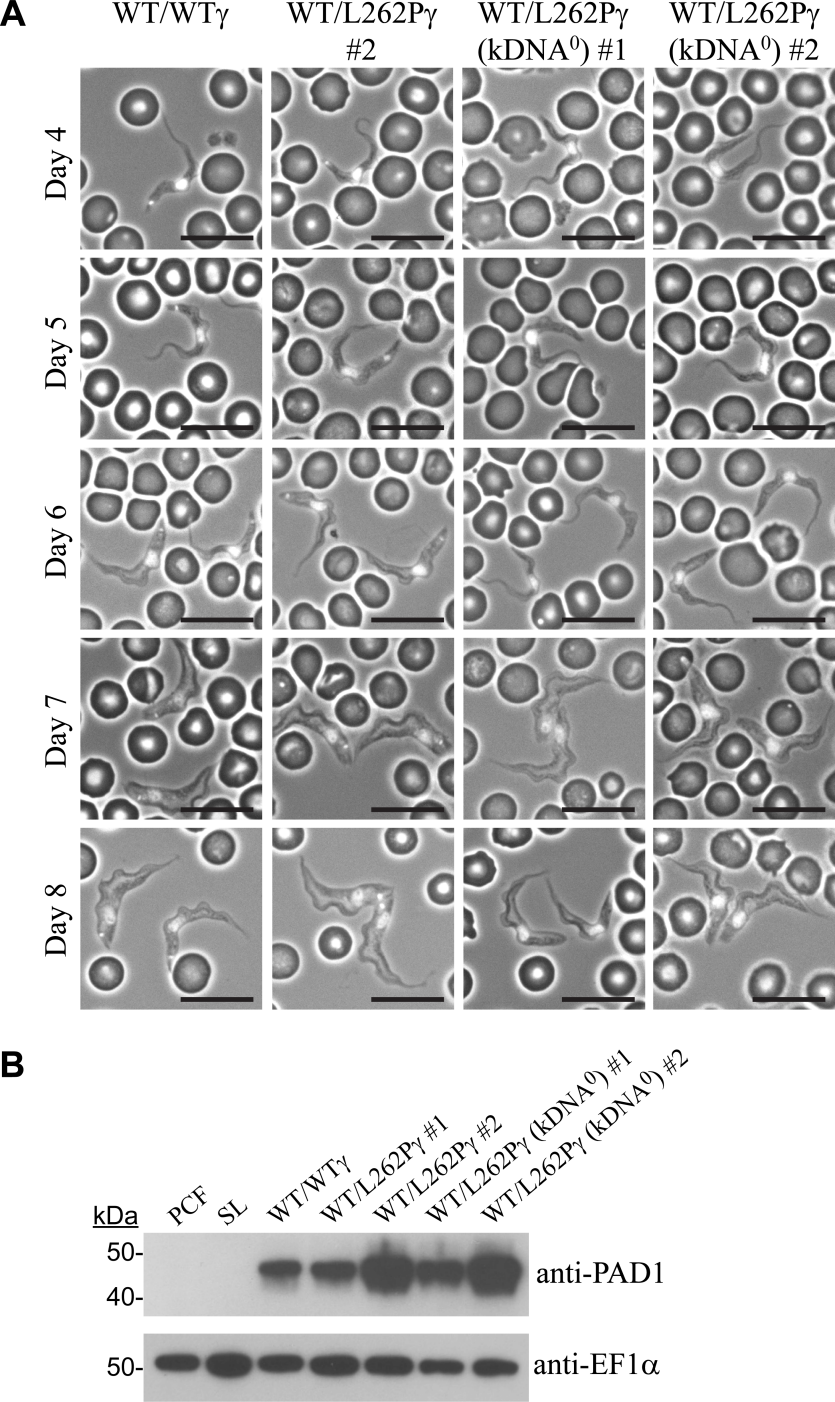
Parasite kDNA is not required for stumpy formation. (A) Representative phase contrast images showing the morphology of cells at days 4–8 of *in vivo* infection. Corresponding parasitaemia graphs are shown in Fig 2. Blood smears were taken daily and DAPI-stained, allowing positioning of the nucleus and kDNA to also be assessed. Scale bars represent 10 μm. (B) Immunoblot analysis of expression of the stumpy-specific protein PAD1. Cells were harvested at peak parasitaemia (day 6). 2x10^6^ cells of each cell line were loaded per lane. PCF = procyclic form Lister 427 29.13; SL = slender BSF of parental EATRO 1125 AnTat1.1 90:13 cell line.

To test the capacity for differentiation to stumpy forms, we infected mice of strain MF1 with cell lines WT/WTγ, WT/L262Pγ #2 and WT/L262Pγ (kDNA^0^ #1 and #2) via IP injection. Accurate measures of parasitaemia level and morphology during the first peak of infection were recorded over time for each cell line in four replicate infections. Parasites that were morphologically stumpy were seen in all infections as they progressed (Fig 1A, days 7–8) and were found to express the stumpy-specific protein PAD1 [35] (Fig 1B). This demonstrated that kDNA^0^ populations were capable of generating stumpy forms.

### Lack of kDNA reduces persistence of stumpy parasites *in vivo*

We next carried out a detailed comparison between cell lines in terms of the efficiency of the slender to stumpy transition, and the length of time that the parasitaemia was maintained, to judge the effect of kDNA loss on the *in vivo* dynamics of a mouse infection.

**Fig 2:**
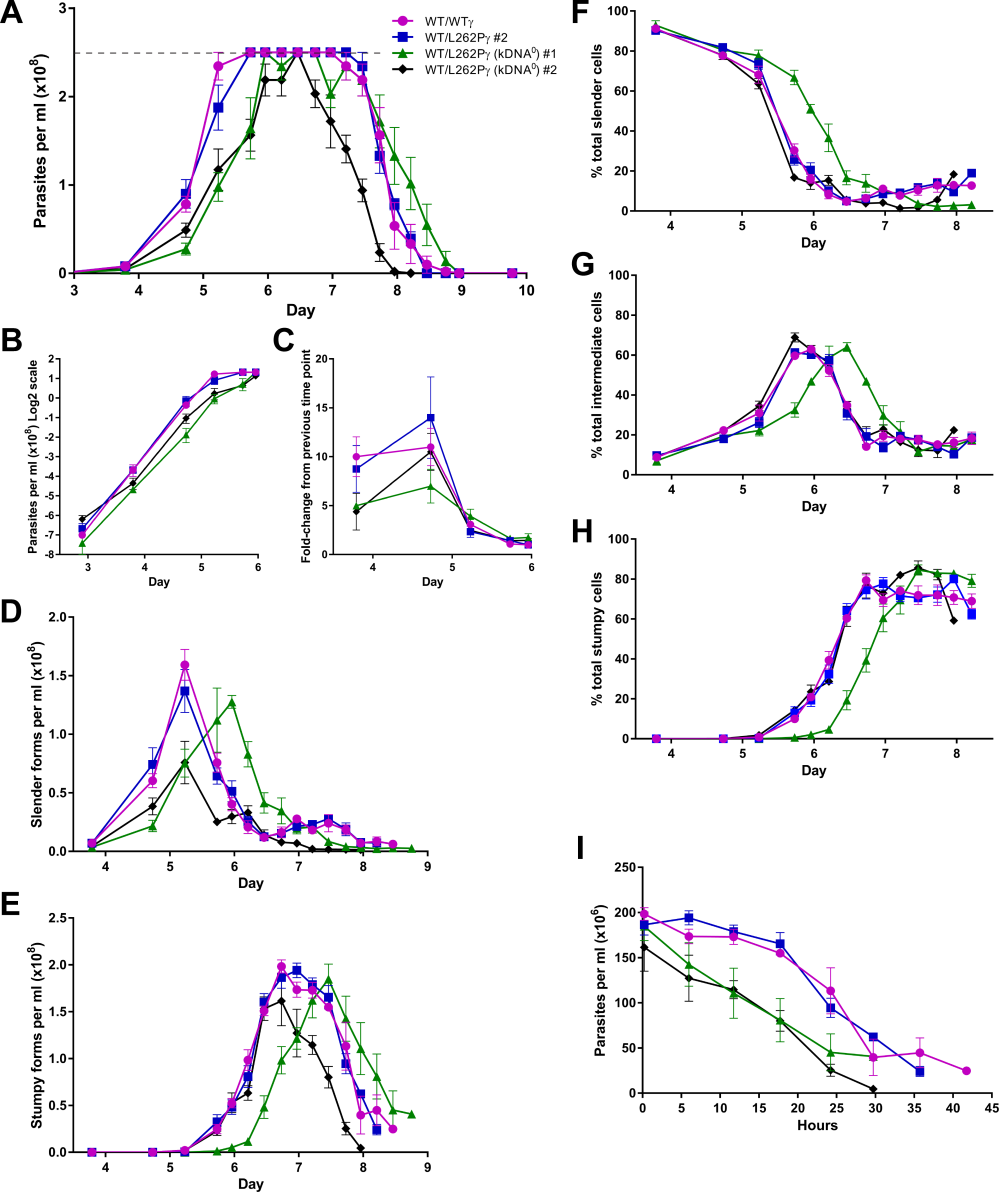
Stumpy forms lacking kDNA persist for a reduced time span during the first peak of parasitaemia. (A) Parasitaemia during mouse infections was measured over time. Mice were infected at day 0, with four mice infected per cell line. Error bars are standard error of the mean (SEM). Tail snips were performed every 6 h from day 4 to day 10 post infection and cell counts were estimated from blood smears. Dashed grey line indicates accurate detection limit via the Rapid Matching method. (B) Data from panel A (up to day 6) plotted on a semi-log scale. (C) Fold-change in parasitaemia between time points (up to day 6). (D-H) The morphological changes occurring during these mouse infections, expressed as cell densities (D, slender forms; E, stumpy forms) or percentages (F, slender cells; G, intermediate cells; H, stumpy cells). Error bars are SEM. The population of cells were scored as having slender, intermediate or stumpy form morphology from DAPI-stained dry blood smears. (I) The levels of parasitaemia of stumpy forms per ml of blood, where t = 0 is the time point where the stumpy number was highest for each cell line. This time point was 6 days and 16 hours for cell lines WT/WTγ, WT/L262Pγ #2 and WT/L262Pγ (kDNA^0^) #2, and 7 days and 12 hours for WT/L262Pγ (kDNA^0^) #1. Error bars are SEM.

Cell lines WT/WTγ and WT/L262Pγ had first peaks of parasitaemia that were very similar to each other (Fig 2A–2E) and to published data [36]. In contrast, we observed three main differences for the kDNA^0^ cell lines: (i) a delayed rise in parasitaemia (Fig 2A–2C); (ii) a more rapid decline in parasitaemia once peak density had been reached (Fig 2A); and (iii) absence of a smaller, second peak in slender form parasitaemia evident in kDNA^+^ cells on days 7–8 post infection (Fig 2D).

Both kDNA^0^ clones showed a delayed rise in cell numbers (Fig 2A, days 3-6), at least in part caused by a slower growth rate up to day 4 (Fig 2B and 2C), suggesting that a lack of kDNA affects the parasite’s ability to proliferate *in vivo* and/or to persist during the transitions they undergo through a mouse infection. To investigate this observation further, we compared the rates of differentiation to intermediate and stumpy cells (Fig 2F–2H). Here, no consistent differences were observed between kDNA^0^ and kDNA^+^ cells. At peak parasitaemia, populations of all cell lines consisted of 80–90% stumpy cells, again demonstrating that kDNA is not required for the differentiation of *T*. *brucei* from slender to stumpy forms. Although differentiation of kDNA^0^ cell line #1 was delayed by approximately half a day, this was not the case for the other kDNA^0^ cell line and therefore unlikely to be a consequence of kDNA loss. The slower growth (and therefore the delayed rise) of these cells could therefore be due to longer cell-cycle times or an increased death rate.

**Fig 3:**
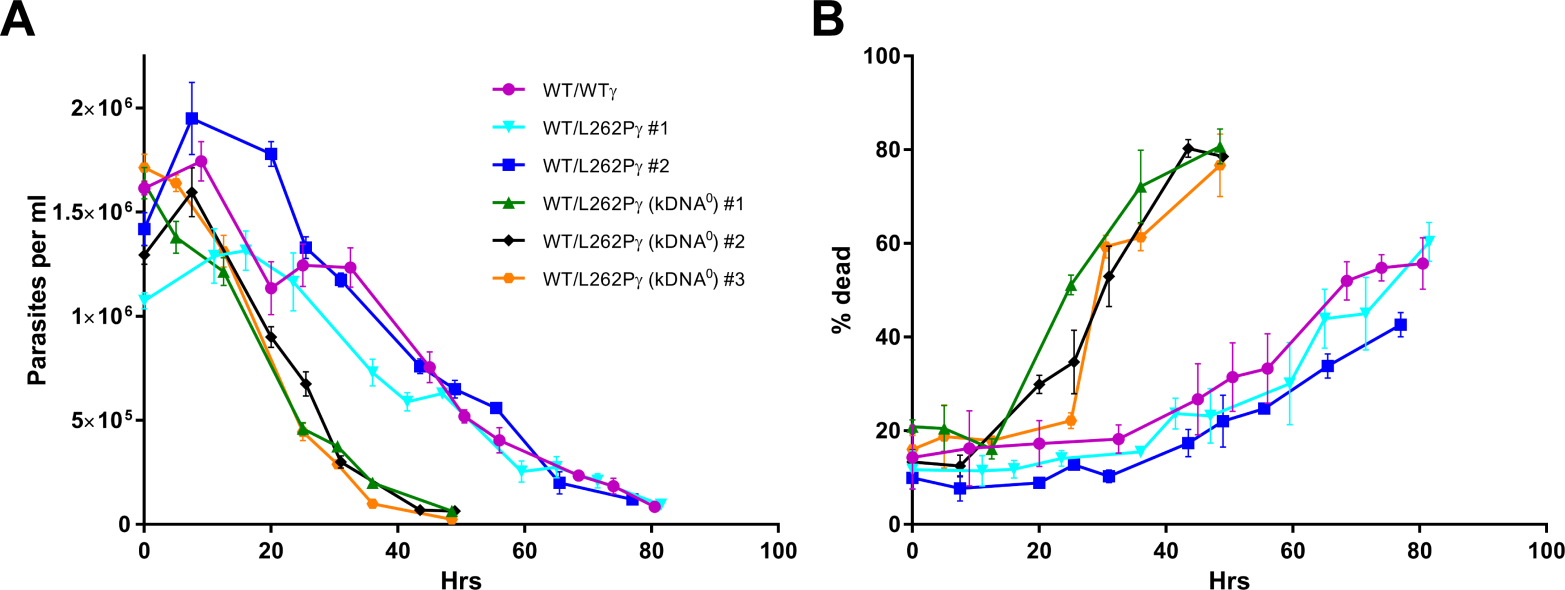
Stumpy form parasites lacking kDNA have a reduced life span *in vitro*. (A) *T*. *brucei* stumpy forms were harvested from mice, purified from blood and cultured in HMI-9 medium containing 50 μM DFMO to suppress slender form growth. Every 8 h cell numbers were determined in a Coulter particle counter. Cell line WT/L262Pγ (kDNA^0^) #3 had lost its kinetoplast spontaneously, without acriflavine treatment. (B) At every time point shown in panel A, 1x10^6^ cells were stained with CFDA-SE and analysed by flow cytometry to determine the percentage of cells that were dead. The gating strategy is shown in supplementary data. Number of replicates n = 3, with error bars showing SEM.

Having reached maximum parasitaemia, total cell numbers for kDNA^0^ parasites declined more rapidly than for kDNA^+^ parasites (Fig 2A, days 6–9). When we assessed stumpy form densities, it was evident that kDNA^0^ stumpy cells maintained high densities for a shorter period of time than kDNA^+^ stumpy cells (Fig 2E and 2I). To investigate this further, we assessed the lifespan of stumpy forms *in vitro*. In this experiment we included a third kDNA^0^ cell line (#3) that had lost its kDNA spontaneously to address the possibility that stumpy lifespan might have been affected by any non-kinetoplast related mutagenic effects of acriflavine. We harvested populations enriched for the stumpy form parasites from mice and incubated them in HMI-9 medium in the presence of the cytostatic agent α-difluoromethylornithine (DFMO); this prevents contaminating slender cells from proliferating [37–39]. We sampled cells every 8 h and determined numbers with a particle counter. We also stained cells with carboxyfluorescein diacetate succinimidyl ester (CFDA-SE) to analyse the proportion of dead cells over time. Consistent with what we observed *in vivo* (Fig 2I), cell numbers for kDNA^0^ stumpy cells dropped more quickly for kDNA^0^ than for kDNA^+^ stumpy cells (Fig 3A), with kDNA^0^ stumpy cells reaching a threshold of 50% dead cells 40–50 h earlier than the kDNA^+^ populations (Fig 3B). All kDNA^0^ cell lines behaved in a very similar manner, confirming that the decrease in stumpy life span was not due to secondary mutations caused by acriflavine treatment.

Finally, kDNA^+^ cells had a second peak in slender parasitaemia around days 7–8 (Fig 2D). This second peak was absent in kDNA^0^ cells. The presence of the second peak in slender density in kDNA^+^ parasites was confirmed by quantifying the percentage of cells in G_2_ phase of the cell cycle in samples taken across the time course, using flow cytometry (S2 Fig).

In summary, our mouse infection data demonstrated differences in the within-host infection dynamics for *T*. *brucei* parasites with and without a kinetoplast. Most importantly, stumpy cells lacking kDNA had a reduced life span.

### Optimisation of a mathematical model for *T*. *brucei* infections

Mathematical models allow complex biological systems to be deconstructed into individual components and parameters, and as such are suitable for quantitation, hypothesis generation and testing. We used an existing mathematical model for *T*. *brucei* infection dynamics [36] to interpret the experimentally obtained data presented in Fig 2 and to provide us with testable hypotheses as to the differences in the infection dynamics observed between kDNA^+^ and kDNA^0^ cells.

**Fig 4:**
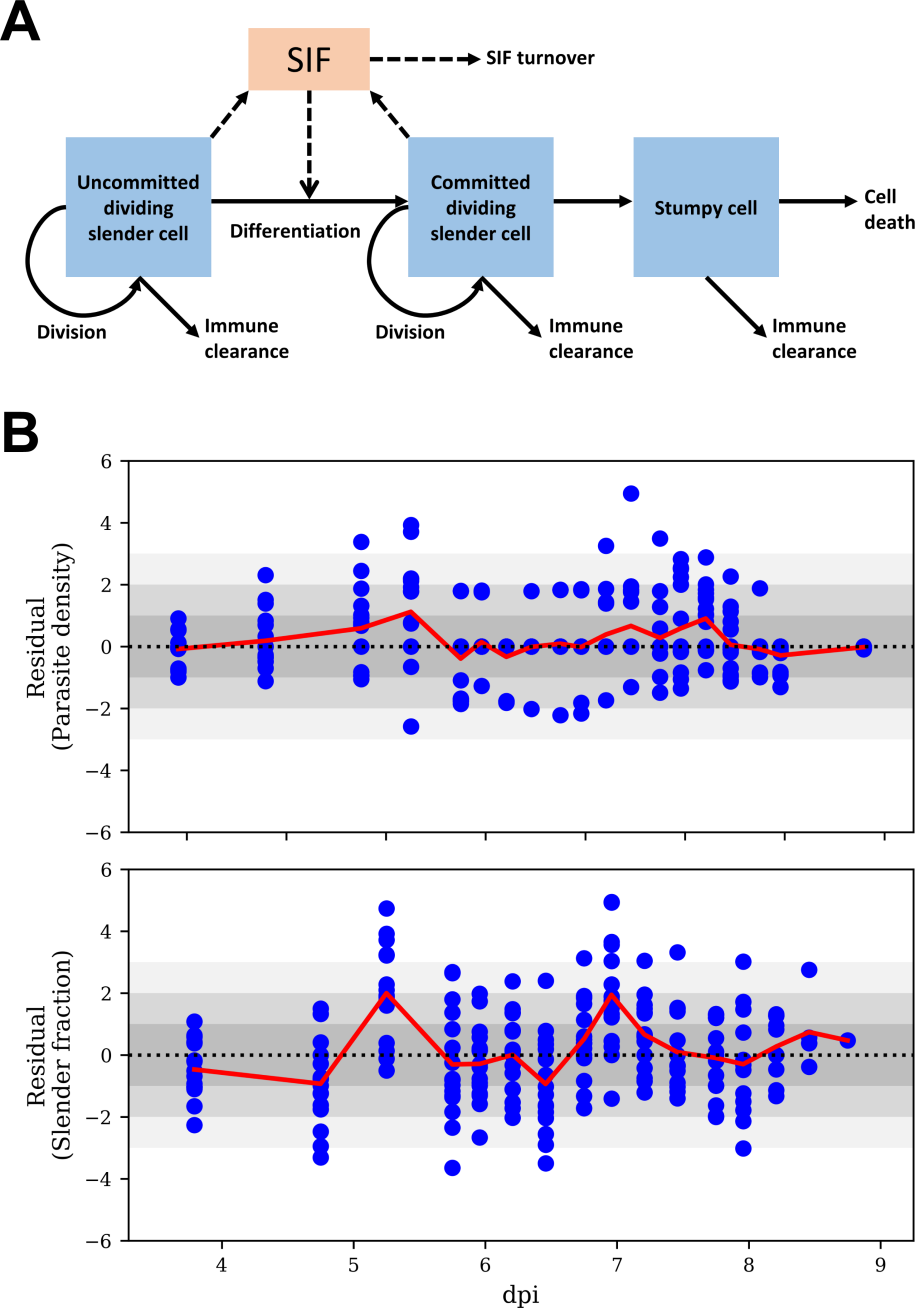
The mathematical model for *T*. *brucei* infection dynamics. (A) Schematic of the mathematical model. Slender cells can become committed to differentiation via a SIF dependent route, proportional to SIF concentration, and a SIF independent route. SIF is produced by both committed and non-committed slender forms, and is cleared over time. The concentration of each cell type depends on the replication rate (applicable to slender forms only), the immune clearance rate, the lifespan of that cell type and the differentiation rate (applicable to slender forms only). (B) Standardised residuals (blue circles) of parasite density and slender fraction, by time (dpi, days post infection), of the model fits with SIF-dependent and -independent differentiation to all mice. Under a true model standardised residuals have an approximately standard normal distribution (i.e., zero mean and unit standard deviation (SD)). Inadequate fit of a model is indicated by its residuals deviating from a standard normal distribution (such as residuals further than ~3 SD from zero, represented by the lightest grey shading, or a set of residuals consistently above or below zero. The red line shows the average, across all mice, of the residuals at a particular time point.

The cell types in this model are (i) non-committed replicating slender cells (i.e. slender cells not yet committed to stumpy formation), (ii) committed replicating slender cells, and (iii) non-proliferating differentiated cells, including both stumpy and intermediate cells (Fig 4A). We first compared two models: the published model, where the differentiation rate was proportional to SIF concentration [36], and a modified version of this model, where slender to stumpy differentiation rates are additionally influenced by a SIF-independent differentiation term [40]. The latter reflects a constant background level of slender form differentiation, independent of the concentration of SIF. Hence, each slender cell has a fixed probability of differentiating per cell cycle independently of SIF. The two terms are summed, with both terms acting to affect differentiation, with the SIF-dependent term only having a significant effect at high slender form concentrations due to the accumulation of high SIF levels. We inferred model parameter estimates (mean and confidence interval) by fitting both models to the data for all 16 mice using a Bayesian MC-MC method (S3 Fig).

We next assessed the fit of each model by residual analysis (Fig 4B and S4A Fig). In the model containing SIF-independent differentiation, there was a lower number of outlying residuals, with most within the 95% predictive interval more of the time than in the model containing only SIF-dependent differentiation. This difference was apparent for the slender proportion on day 4. The model with additional SIF-independent differentiation captured the drop in slender proportion from 100% to 90% by day 4 (Fig 4B; S3 Fig, panels A, C, E and G, dark blue curves). In contrast, the model with only SIF-dependent differentiation could not capture this drop as there was an insufficient accumulation of SIF by this time to induce such a large amount of differentiation to stumpy forms, resulting in larger residuals (S4A Fig; S3 Fig, panels B, D, F and H, dark blue curves). Hence, this model overestimated the slender proportion on day 4. We also assessed the models by the Akaike information criterion (AIC), which measures the quality of a fit of a mathematical model to a set of data, taking into account the goodness of fit and the number of parameters estimated in the model [41]. The models with and without SIF-independent differentiation had AIC values of 2659 and 3033, respectively, hence the former is preferred as it has the lower AIC. When we reanalysed the infection data from an earlier study [36] by including the additional SIF-independent term, that model was also preferred when mathematically assessed by the AIC (S4B Fig).

In conclusion, the mathematical model for within-host infection dynamics of *T*. *brucei* provided a better fit to experimental data when it included an additional term for SIF-independent slender-to-stumpy differentiation. This is consistent with the recent identification of a quorum sensing-independent path to stumpy development in this parasite [42].

### Infection dynamics as predicted from improved mathematical modelling

We next used the optimised mathematical model to identify and quantitate the parameters predicted to be responsible for these differences between kDNA^+^ and kDNA^0^ cell lines. This resulted in two key observations.

**Fig 5:**
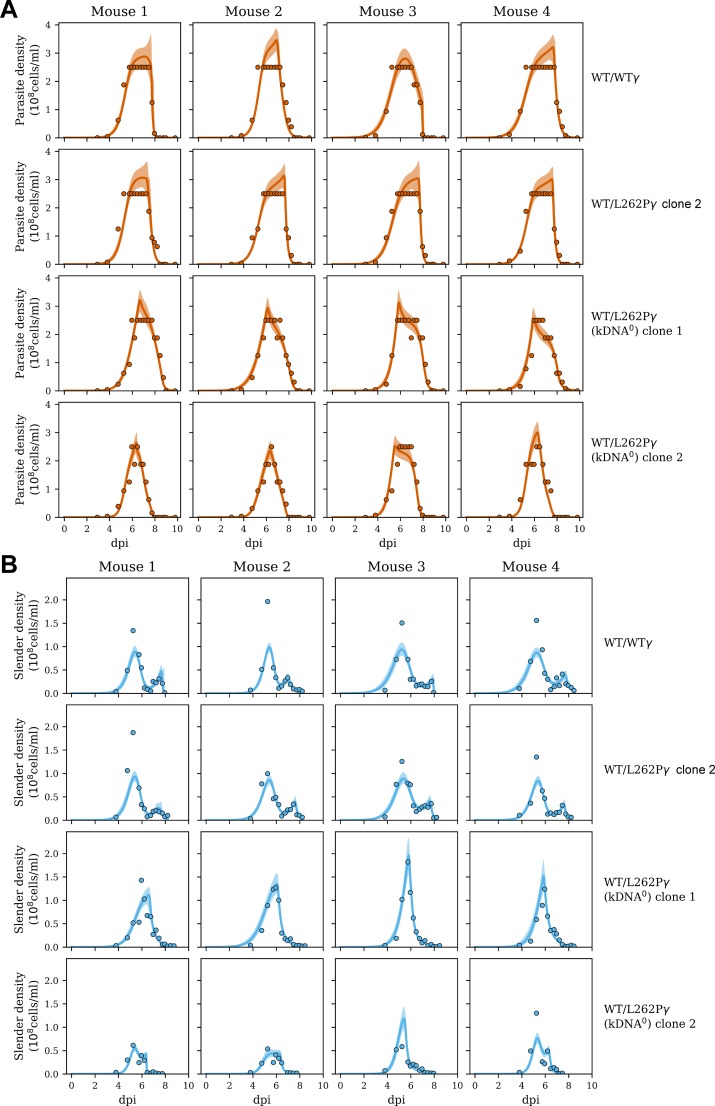
Comparison of mathematical model and data from mouse infections. The data is represented by filled dots. The coloured lines represent median fits of the model (including terms for both SIF-dependent and -independent differentiation); the shaded regions indicate 95% predictive intervals, where 95% of future data would be predicted to lie according to the model and the data already observed. (A) Levels of overall parasitaemia over the course of the experiment. (B) Levels of parasitaemia for slender forms only. The complete analysis is shown in S3 Fig; dpi, days post infection.

First, infection with kDNA^+^ parasites resulted in a broader peak of high cell density than infection with kDNA^0^ parasites (Fig 5A). The model predicts that the rise in parasitaemia levels off due to SIF-induced differentiation to the stumpy form: as slender forms proliferate, SIF begins to rise (S3 Fig, pink curves), which increases the rate of differentiation, and stumpy forms begin to emerge (S3 Fig, light orange curves). As the number of slender forms decrease, SIF concentration falls, causing the differentiation rate to fall. Stumpy cells disappear due to an intrinsically limited life span or immune clearance (Fig 4A). The estimates for total committed lifespan of kDNA^+^ and kDNA^0^ cells were 72–77 h and 41–51 h, respectively (Table 2). The total committed lifespan can be broken down into duration of the committed slender form and the duration of the stumpy form. Committed slender kDNA^+^ cells were estimated to survive longer than kDNA^0^ cells; they were predicted to have gone through at least one further cell cycle division than kDNA^0^ cells before they entered cell cycle arrest as the intermediate form (Table 2, ‘Committed slender replications’). Stumpy forms with kDNA lived on average for 56–62 h, whereas for kDNA^0^ cells the calculated average stumpy lifespan was predicted to be considerably lower, 36–49 h. Interestingly, the model predicted a clear difference between the immune responses to kDNA^+^ and kDNA^0^ stumpy cells. While the model estimated similar and consistent clearance rates for WT/WTγ and WT/L262Pγ stumpy cells, at ~20% per hour (Table 2), it estimated that immune clearance was not required to explain the disappearance of kDNA^0^ stumpy cells. We note that immune response against trypanosomes, although multifactorial, highly complex and incompletely understood, is represented by a simple step function in our model. There is insufficient data to support more realistic representations of the immune response. Nonetheless, our model fitted the experimental data very well and predicted that the narrower peak of high parasitaemia in kDNA^0^ parasites (see Fig 5A) was largely due to accelerated cell death of stumpy cells lacking kDNA.

**Table 2:** Average parameter estimates for all cell lines. Shown are parameter estimates determined from the model with both SIF-dependent and -independent differentiation terms.

Parameter	Mean +/- SEM			
	WT/WTγ	WT/L262Pγ #2	WT/L262Pγ (kDNA^0^) #1	WT/L262Pγ (kDNA^0^) #2
**Slender doubling time (h)**	6.7 ± 0.5	6.8 ± 0.3	6.9 ± 0.3	4.9 ± 0.1
**SIF-dependent differentiation rate ((10**^**−9**^ **cells/ml)/h)**	2.1 ± 0.1	2.2 ± 0.1	0.56 ± 0.08	2.3 ± 0.4
**Start time of immune response (h)**	179 ± 4	180 ± 1	146 ± 4	144 ± 5
**Duration of committed slender form (h)**	15.2 ± 1.3	15.2 ± 0.1	2.3 ± 1.3	5.7 ± 1.4
**Duration of stumpy form (h)**	56. ± 5	62 ± 1	49 ± 1	36 ± 4
**Total committed lifespan (h)**	72 ± 5	77 ± 1	51 ± 2	41 ± 4
**Immune clearance rate of slender forms (/h)**	0.36 ± 0.11	0.24 ± 0.01	0.16 ± 0.002	0.20 ± 0.01
**Immune clearance rate of stumpy forms (/h)**	0.24 ± 0.08	0.17 ± 0.02	0	0
**Committed slender replications**	2.26 ± 0.05	2.25 ± 0.09	0.40 ± 0.20	1.17 ± 0.29
**Probability of SIF independent differentiation per cell cycle (%)**	5.0 ± 0.5	4.6 ± 0.6	5.8 ± 0.8	5.9 ± 0.9

Second, in kDNA^+^ cells, a second peak in parasitaemia emerged around day 7, when a reduced SIF concentration allowed slender cells to proliferate again (Fig 5B). According to the model, the density fell again due to onset of immune killing (S3A and S3C Fig, yellow curves). Without immune killing the model predicts a continued rise in slender density to a much higher level. The absence of this second peak in kDNA^0^ parasites (Fig 5B) was explained by our model with an onset of immune killing about 1.5 days earlier (S3E and S3G Fig, yellow curves; Table 2, ‘Start time of immune response’), which completely suppressed the second rise in parasitaemia.

In summary, an optimised mathematical model for within-host infection dynamics that included an additional SIF-independent parameter for slender-to-stumpy differentiation provided a very good fit to experimental data and captured experimentally observed differences between kDNA^+^ and kDNA^0^ parasites. A narrower peak of high parasitaemia in the latter was predicted to be largely due to accelerated cell death of stumpy forms lacking kDNA.

### Absence of kDNA is associated with loss of ΔΨm in stumpy forms

The shortened lifespan of kDNA^0^ stumpy forms (Fig 2 and Fig 3) pointed to loss of critical mitochondrial functions. A hallmark of functional mitochondria is the presence of ΔΨm. In BSF *T*. *brucei* ΔΨm is primarily generated by ATP hydrolysis-driven proton pumping of the F_1_F_O_-ATPase, whereas PCF *T*. *brucei* generate ΔΨm by proton pumping of respiratory complexes III and IV and, potentially, cI. It is not clear how the ΔΨm is generated in stumpy forms, although it has been reported to be sensitive to cI inhibitors but insensitive to the F_1_F_O_-ATPase inhibitor oligomycin [14].

**Fig 6:**
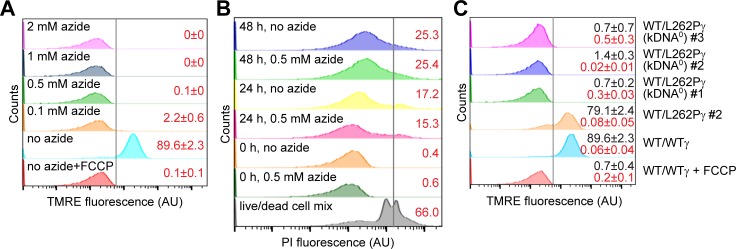
Stumpy forms without kDNA lack ΔΨm. For all experiments, stumpy cells were purified from blood and incubated in HMI-9 medium with 10% (v/v) FCS. Specific experimental conditions were as indicated. AU, arbitrary units. (A) The effect of F_1_-ATPase inhibitor azide on the ΔΨm of WT/WTγ stumpy form cells. Where indicated, cells were pre-incubated with azide for 2 h before tetramethylrhodamine ethyl ester (TMRE, 100 nM) was added to the medium. ΔΨm was measured by flow cytometry (peak excitation 549 nm, peak emission 575 nm). Representative analyses are shown, n = 3. The numbers in red on the right indicate the % of cells (±SD) determined to be ‘ΔΨm positive’. The ‘ΔΨm negative’ gate (black vertical line) was set to include all counts up to the maximum fluorescence detected for WT/WTγ cells incubated with an uncoupler, carbonyl cyanide-4-(trifluoromethoxy)phenylhydrazone (FCCP, 20 mM). All counts to the right of this gate were defined as ‘ΔΨm positive’. (B) Effect of azide on viability of WT/WTγ stumpy cells. Cells were incubated in HMI-9 medium for 0, 24 or 48 h, +/- 0.5 mM azide. At each time point, 1x10^6^ cells were stained with propidium iodide (PI) and TMRE, and analysed by flow cytometry. The numbers in red on the right indicate the average % dead cells within each population; n = 2. The black vertical line indicates the gate for live/dead staining by PI, established as shown in S5B Fig. The TMRE assays for these samples are shown in S5C Fig. (C) The ΔΨm of stumpy form cells with genotypes as labelled, measured as described for panel A, and indicated as % ‘ΔΨm positive’ cells (average ±SD) by the black value on top. The numbers in red below give the % of dead cells within each cell population (average ±SD), measured by PI staining as described for panel B.

To explore the role of kDNA in maintaining ΔΨm in stumpy forms, we harvested parasites from all five cell lines (WT/WTγ, WT/L262Pγ, and the three WT/L262Pγ kDNA^0^ cell lines) from infected mice at maximum parasitaemia, with a proportion of approximately 90% stumpy cells (S5A Fig), and stained them with the ΔΨm probe tetramethylrhodamine ethyl ester (TMRE) under various experimental conditions. First, we assessed ΔΨm in WT/WTγ cells in the presence or absence of azide, a specific inhibitor of the F_1_ moiety that disrupts ΔΨm production in slender BSF cells and kills these cells in 36–48 h [23]. Treatment with 0.1–2 mM azide completely abolished ΔΨm (Fig 6A), indicating that F_1_ has an essential role in generating the ΔΨm in the stumpy form. Although treatment with 0.5 mM azide eliminated ΔΨm, it did not reduce the viability of stumpy forms: in the absence of azide, the percentage of dead cells in the population increased from 0.4% to 17.2% after 24 h, and to 25.3% after 48 h, and these percentages were not significantly increased in the presence of azide (Fig 6B and S5C Fig). This suggested that maintaining ΔΨm is not critical for the viability of stumpy forms.

The role of the F_1_ ATPase in generating ΔΨm could be direct, as described above, or indirect, as in slender BSF kDNA^0^ cells. In the latter, the ATP/ADP carrier (AAC) acts to generate ΔΨm via the electrogenic exchange of matrix ADP^3-^ for cytosolic ATP^4-^. F_1_ acts independently of F_O_ by hydrolysing ATP^4-^ to maintain an ATP/ADP ratio across the inner mitochondrial membrane that can sustain AAC activity [16,23]. We investigated whether this F_O_-independent pathway can function in stumpy forms by assessing ΔΨm in WT/L262Pγ (kDNA^0^) cells purified from mice. Live, freshly isolated kDNA^0^ stumpy cells were found to not have a ΔΨm (Fig 6C), demonstrating that the kDNA-encoded *a* subunit of the F_o_-proton pore is required for ΔΨm generation and that this requirement cannot be circumvented by the L262Pγ mutation. Hence, the alternative, F_O_-independent mechanism of ΔΨm generation that functions in kDNA^0^ slender *T*. *brucei* and in subspecies *T*. *b*. *evansi* and *T*. *b*. *equiperdum* cannot operate in stumpy forms.

### kDNA^0^ stumpy forms cannot use α-KG to sustain viability

The stumpy life cycle stage is preadapted to differentiation to the PCF in the midgut of the tsetse fly. Stumpy forms can use glycolysis or, alternatively, mitochondrial catabolism of α-KG as energy sources [9,10,13], which reflects the shift in metabolism towards the glucose-deficient environment of the tsetse midgut.

**Fig 7:**
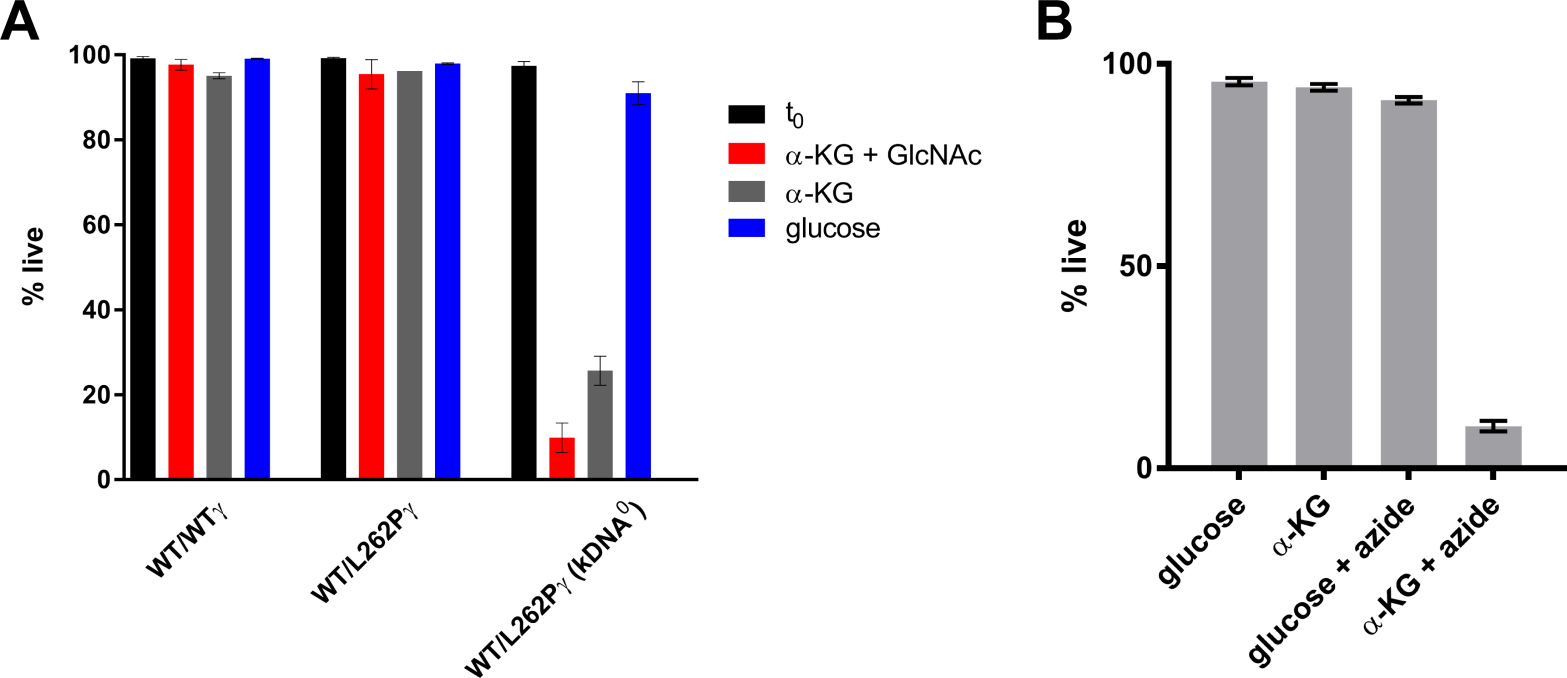
Stumpy forms without kDNA die rapidly when α-KG is the main carbon source. (A) Stumpy forms of the genotypes indicated were harvested, purified from blood and placed in Creek’s minimal medium (CMM) with 10% (v/v) FCS, supplemented with either glucose (blue bars) or α-KG (grey bars). N-acetyl glucosamine (GlcNAc, 50 mM) was added to one set of experiments to reduce uptake of residual glucose from FCS (red bars). Cells were stained with PI and the % of live cells was assessed by flow cytometry before (t_0_; black) and 24 h after the start of the experiment; n = 3 for each cell line; all three kDNA^0^ cell lines were assessed and data averaged (total n = 9). The gating strategy is shown in S5B Fig. (B) Quantification of dead cells within WT/WTγ cell populations after 24 h in CMM supplemented with either glucose (25 mM) or α-KG (25 mM), with or without azide (0.5 mM). Cells were stained with PI and analysed by flow cytometry, the gating strategy is shown in S5B Fig. Shown are average values ±SD; n = 3.

We assessed the ability of kDNA^0^ stumpy forms to survive in the presence of glucose or α-KG. Although kDNA^0^ stumpy forms in the presence of glucose showed normal viability after 24 h, more than 70% of cells had died after 24 h of incubation with α-KG as sole major carbon source (Fig 7A). When α-KG was provided in addition to glucose it had little, if any, detrimental effects on kDNA^0^ cells (S5D Fig). The addition of N-acetyl glucosamine (GlcNAc), a non-metabolized glucose analog, to prevent uptake of residual glucose present in fetal calf serum (FCS) [43] further reduced the number of surviving cells (Fig 7A). The viability of kDNA^+^ control cells was comparable for medium with α-KG vs. glucose as main carbon source, and addition of GlcNAc to medium with α-KG had no negative effects on WT/WTγ cells (S5D Fig), confirming that GlcNAc only interferes with glucose-based energy metabolism. These results demonstrate that, unlike kDNA^+^ stumpy forms, kDNA^0^ parasites are unable to use α-KG to sustain viability. Interestingly, stumpy WT/WTγ cells treated with azide to inhibit generation of a ΔΨm died within 24 h if α-KG was the sole carbon source, but azide had little effect in the presence of glucose (Fig 7B), indicating that ΔΨm may be required for the entry of α-KG into the mitochondrion or for efficient export of mitochondrially produced ATP.

In summary, these experiments suggest that the lack of a ΔΨm in stumpy cells without kDNA precludes the use of α-KG to satisfy the energy needs of these cells.

## Discussion

The mitochondrion plays essential roles in the life cycle and the cell cycle of *T*. *brucei* and is an important target for existing anti-trypanosomatid chemotherapies, but our knowledge of its precise functions in each of the life cycle stages and how these are regulated is far from complete [44]. One gap in knowledge concerns mitochondrial biology, and in particular the role of kDNA-encoded proteins in the so-called stumpy stage, which dominates within-host dynamics and is critical for transmission to the tsetse fly vector [36].

**Fig 8:**
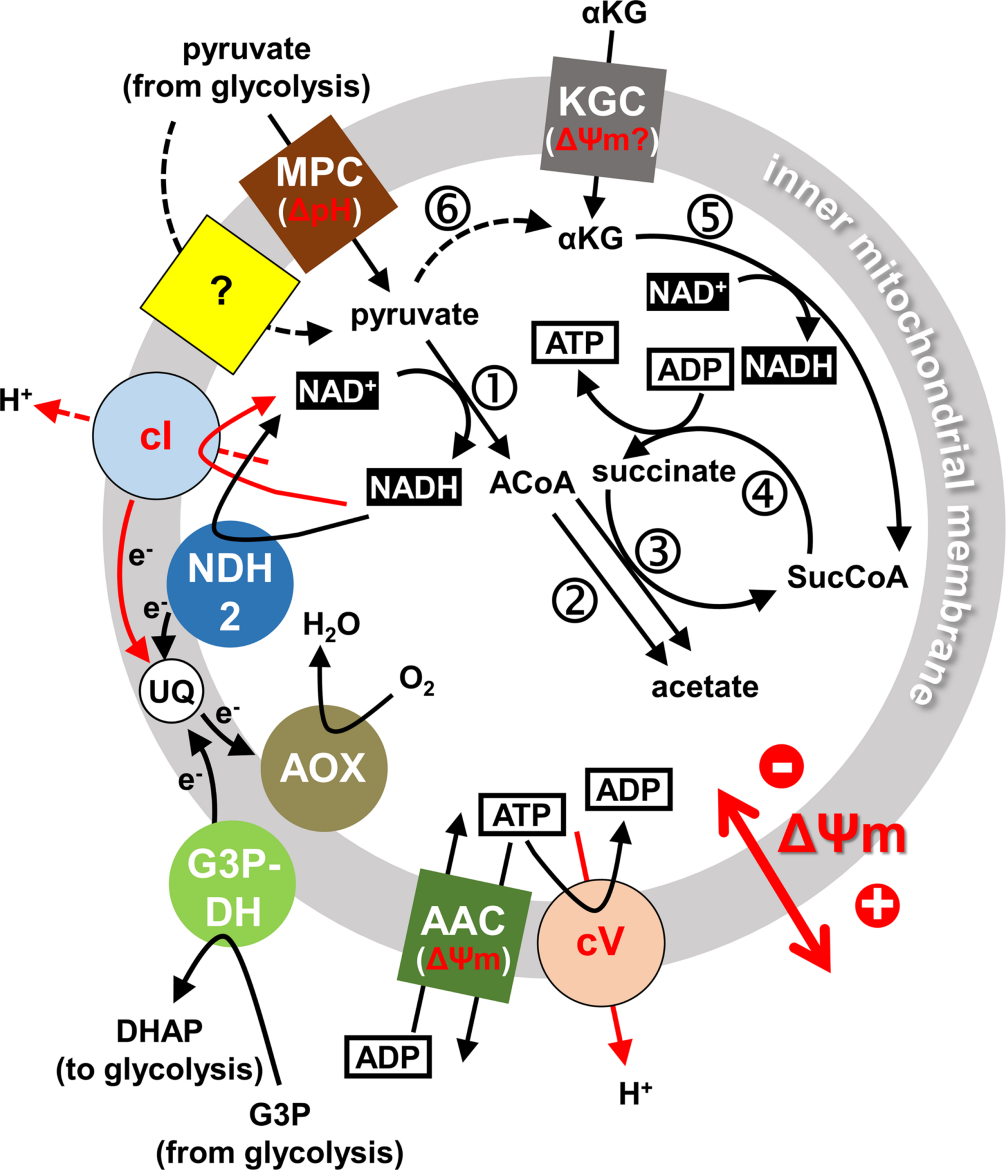
Proposed mitochondrial energy metabolism of stumpy form *T*. *brucei* cells in the bloodstream. Schematic representation of key functions we propose to be involved in energy metabolism of stumpy form *T*. *brucei*, based on data presented in this work and in earlier studies, as cited in the text. Note that energy metabolism in other compartments such as adipose tissue or skin will very likely be different. Transporters in the inner mitochondrial membrane are shown as coloured squares (MPC, mitochondrial pyruvate carrier; KGC, α-KG carrier; AAC, ATP/ADP carrier). A two-subunit mitochondrial pyruvate carrier, MPC1/2, presumably driven by proton symport, has been identified in *T*. *brucei*, but functional studies concluded that at least one additional mitochondrial pyruvate transporter must be present [74], indicated here by a yellow square with a question mark. Enzymes or enzyme complexes associated with the inner membrane are shown as coloured circles (cI, NADH:ubiquinone oxidoreductase; cV, F_1_F_O_-ATPase, or respiratory complex V; G3P-DH, glycerol-3-phosphate dehydrogenase; AOX, alternative oxidase; NDH2, type 2 NADH dehydrogenase). Functions that directly depend on kDNA-encoded proteins are indicated by red letters and arrows. Key metabolic reactions in the mitochondrial matrix are indicated by numbers in circles: 1, pyruvate dehydrogenase; 2, acetyl-CoA thioesterase; 3, ASCT; 4, SCoAS; 5, α-KG dehydrogenase complex; 6, L-alanine aminotransferase (co-substrate glutamate and co-product alanine omitted for simplicity). Other abbreviations: UQ, ubiquinone; G3P, glycerol-3-phosphate; DHAP, dihydroxyacetone phosphate; ACoA, acetyl-CoA; SucCoA, succinyl-CoA).

To provide insight into these questions we have introduced a subunit γ allele with the L262P mutation (‘L262Pγ’) into a pleomorphic (i.e. differentiation competent) *T*. *brucei brucei* cell line by *in situ* replacement of one of the endogenous alleles. This mutation enables slender BSF *T*. *brucei* to proliferate without kDNA *in vitro* and *in vivo* [16]. From such heterozygous WT/L262Pγ cell lines we obtained kDNA^0^ mutants by acriflavine exposure or through accidental loss of the kinetoplast. We then studied within-host dynamics in a mouse model, interpreted the data with the help of mathematical modelling and investigated the molecular basis for the observed phenotypes with cell physiological assays. Our study shows that parasite kDNA is critical for full viability of the transmissible stumpy stage and suggests a model for mitochondrial energy metabolism in these forms (Fig 8).

### Slender BSF *T*. *brucei* lacking kDNA can differentiate into stumpy forms

Our mouse infections with cell lines with the genotypes WT/WTγ, WT/L262Pγ and WT/L262Pγ (kDNA^0^) and quantification of within-host dynamics using mathematical modelling showed that lack of kDNA did not affect the rate of differentiation into stumpy forms. The morphological changes we observed during slender to stumpy differentiation were similar for kDNA^+^ and kDNA^0^ cells. These results confirm conclusions from an earlier study that had investigated differentiation of kDNA-depleted *T*. *brucei* cells obtained by treatment with acriflavine for 24 h [18], but that study could not rule out that some kDNA-encoded factors had persisted after treatment.

As kDNA^0^ slender BSF *T*. *brucei* are able to transition to the stumpy form as efficiently as cells that have their kDNA intact, we conclude that the absence of kDNA is not the primary reason why the dyskinetoplastic subspecies *T*. *b*. *evansi* and *T*. *b*. *equiperdum* are generally monomorphic [29,31]. A different molecular mechanism must therefore prevent naturally occurring kDNA^0^ or kDNA^-^ *T*. *brucei* subspecies from differentiating to the stumpy life cycle form, such as loss of function in components of the SIF secretion or stumpy induction pathways [1], in a fashion similar to monomorphic *T*. *b*. *brucei* BSF forms [2]. SIF-dependent differentiation to the stumpy form places a limit on the parasitaemia level, presumably in part to extend the lifespan of the host [1]. As the probability of mechanical transmission of trypanosomes increases with the levels of parasitaemia in the blood [45], preventing slender to stumpy differentiation could thus have been a key event in the evolution of *T*. *b*. *evansi*, as has been discussed elsewhere [46,47]. Genome sequences from a number of *T*. *b*. *evansi* isolates are now available [26,48] and could be mined for candidate mutations in differentiation pathways.

A few *T*. *b*. *evansi* strains have historically been reported to have some limited capacity to produce stumpy forms (for example [30]); this could be due to SIF-independent background differentiation or residual and inefficient SIF-dependent differentiation. We found that a mathematical model including SIF-independent differentiation provided a better fit to the experimental data from both the current study and a previous infection study [36] than a model only including SIF-dependent differentiation. A recent study provided biological evidence for SIF-independent differentiation in trypanosome infections [42] and there is emerging evidence for a background level of random differentiation in *Plasmodium* and *Theileria* infections [49–51]. Thus, our study supports the view that stochastic, low level differentiation events occurring in parallel to a signal transduction-type of differentiation could be a more broadly conserved aspect of infections with protist parasites.

### Loss of kDNA affects within-host infection dynamics of *T*. *brucei*

Although kDNA^0^ *T*. *brucei* differentiated into stumpy forms with the same efficiency as control cells, we observed some important differences in other aspects of their within-host dynamics. Firstly, kDNA^0^ cells showed a slightly lower growth rate up to day 4 of infection, resulting in a delayed rise in cell numbers leading up to the first peak of parasitaemia. In contrast, growth rates of kDNA^0^ and kDNA^+^ parasites were very similar when cultured in rich medium *in vitro*. Potential explanations are that kDNA^0^ parasites are more affected by the more limiting growth conditions in the host environment, or that they are more sensitive to attack by the host’s immune system, or both. Our mathematical model predicts that the immune response does not significantly affect infection dynamics until day 6 or 7 (although there appear to be differences for kDNA^0^ vs. kDNA^+^ parasites, see below), but as the action of the immune system during a trypanosome infection is not fully understood, our modelling of this aspect is necessarily an oversimplification. Understanding these differences will require further investigation, for example by comparing growth rates in minimal medium [52] and by investigating infection dynamics in immunosuppressed mice.

Secondly, a second peak in slender parasitaemia was evident in kDNA^+^ cells around days 7–8 post infection, but completely absent in kDNA^0^ cells. According to the mathematical model, this second slender peak in infections with kDNA^+^ cells was due to SIF-dependent differentiation causing slender density, and therefore SIF density, to fall around day 6, allowing the remaining slender cells to begin proliferation rather than entering cell cycle arrest. A strong immune response around day 8 then prevented a further rise in parasitaemia. The model predicts that an earlier onset of immune killing in infections with kDNA^0^ cells was responsible for completely suppressing this second peak in these parasites. This surprising result requires further investigation; we speculate that kDNA^0^ cells could be less efficient at VSG switching or production, allowing more efficient clearance of slender cells in the earlier stage of the infection. Alternatively, kDNA^0^ cells could be less able to access potentially immune privileged body compartments [53,54], or they could swim more slowly or in a different way, preventing the efficient clearance of antibody that is mediated by swimming [55].

Finally, and most importantly, we observed a substantially shorter lifespan of kDNA^0^ stumpy forms. For kDNA^+^ *T*. *brucei* we determined an average value for ‘duration of stumpy form’ of 56–62 h. This is in good agreement with other reports using a mouse infection model [36,38]. There are no reports on stumpy cell lifespan in other hosts, or how it might be affected by parasite distribution in different tissues [53], but our *in vitro* survival assay showed that >90% of kDNA^+^ stumpy cells had perished 70 h after isolation from a mouse, i.e. within a time span comparable to the one observed *in vivo*. In marked contrast, we determined substantially shorter lifespans for kDNA^0^ stumpy cells, both *in vivo* (duration of stumpy forms 36–49 h) as well as *in vitro*. This indicated that the underlying cause was intrinsic to the parasites, rather than due to faster immune clearance.

### Differences in mitochondrial physiology of stumpy stage *T*. *brucei* with and without kDNA

The mechanism of cell death in stumpy forms is not understood, but an early event in programmed cell death in other organisms can be loss of ΔΨm [56–58]. Furthermore, ΔΨm is a key indicator of mitochondrial health [59], it is essential for mitochondrial protein import and other transport processes [60], and its generation in both BSF and PCF *T*. *brucei* depends on kDNA-encoded proteins [23,44].

In the present study we show that the F_1_-ATPase inhibitor azide completely abolished ΔΨm in kDNA^+^ stumpy cells, suggesting its generation by the F_1_F_O_-ATP synthase functioning as a proton pump, as in slender BSF cells [20,21,23,24]. We propose that the switch in directionality of this enzyme from ATPase to ATP synthase activity occurs during the transition from stumpy BSF parasites to PCF parasites. This is also consistent with the increase of the IF1 protein during that transition measured in a recent proteomics study [61]. IF1 (Tb927.10.2970) is a specific inhibitor of the ATP hydrolase activity of the F_1_F_O_-ATP synthase [62] and shows strict developmental regulation in *T*. *brucei*, with repression in slender BSF and expression in PCF [63].

An earlier study had reported that ΔΨm in stumpy forms was sensitive to the cI inhibitor rotenone but insensitive to the F_1_F_O_-ATP synthase inhibitor oligomycin [14]; the authors of that study had concluded that cI generates ΔΨm in stumpy forms, with the F_1_F_O_-ATP synthase acting in ATP synthesis mode, driven by the proton motive force. One possible explanation for this apparent discrepancy is that the relatively high concentration of rotenone used in the earlier study had caused non-specific effects, as has been argued by others [64]. Future studies with genetic mutants for specific subunits of cI and the F1F_O_-ATP synthase in a pleomorphic *T*. *brucei* strain will be required to investigate this apparent discrepancy further.

We did not detect a ΔΨm in kDNA^0^ stumpy cells, indicating that the alternative, F_O_-independent mechanism for generating ΔΨm enabled by the L262Pγ mutation in slender *T*. *brucei* [16] cannot operate in the stumpy life cycle stage. This alternative mechanism depends on electrogenic exchange of matrix ADP^3-^ for cytosolic ATP^4-^ by the AAC and continued ATP hydrolysis by F_1_, perhaps in vicinity of the AAC, to maintain a suitable ATP/ADP ratio across the inner mitochondrial membrane [16,23,65]. Significant mitochondrial ATP production would be expected to thwart this mechanism, and indeed there is evidence for this occurring in stumpy form *T*. *brucei* [10,12–14]. Conceivably this could occur via F_1_F_O_-ATP synthase activity, as mentioned above, or, more consistent with our data, via substrate level phosphorylation involving SCoAS and, depending on the carbon source, ASCT [3,66,67]. Pyruvate from glycolysis can be catabolised by stumpy cells to acetate, with ATP production via the ASCT / SCoAS cycle (Fig 8) [12]. It was also demonstrated that motility of stumpy cells, but not of slender BSF cells, can be sustained *in vitro* with α-KG as sole carbon source [9], with mainly succinate as end product [10] and ATP production via SCoAS (Fig 8). A putative mitochondrial α-KG transporter, termed MCP12 (Tb927.10.12840), has been identified and functionally characterised in *T*. *brucei* [68,69], and a proteomics study reported ~20-fold upregulation of this protein in stumpy cells compared to slender cells [61]. Potentially, pyruvate could be converted to α-KG via L-alanine aminotransferase (Fig 8), an enzyme expressed in BSF and PCF *T*. *brucei* [70]. This step would require glutamate as co-substrate, which could be obtained directly from the medium or via proline catabolism.

We confirmed that kDNA^+^ stumpy cells maintain viability for at least 24 h when incubated in minimal medium supplemented with glucose or α-KG. Nearly 100% of kDNA^0^ stumpy cells survived for at least 24 h when medium was supplemented with glucose, but the survival rate dropped to ~20% when provided with α-KG instead of glucose, and suppressing uptake of residual glucose with GlcNAc resulted in a further drop to less than 10% survivors. We also found that azide, which abolished ΔΨm in kDNA^+^ stumpy cells, prevented survival of these cells in minimal medium supplemented with α-KG, while it did not affect survival in the presence of glucose. At least two scenarios that are not mutually exclusive could explain these results. Firstly, ΔΨm could be required for mitochondrial uptake of α-KG. The transporter identified in *T*. *brucei* was proposed to be an α-KG/malate antiporter [69], analogous to the mammalian enzyme, although this has not yet been confirmed experimentally. In that case α-KG import would not be directly dependent on ΔΨm. The *E*. *coli* enzyme is an α-KG/proton symporter that depends on a proton motive force [71], but its closest homolog in *T*. *brucei* is a myo-inositol/proton symporter in the Golgi [72]. Secondly, α-KG import could be ΔΨm-independent and still drive mitochondrial substrate phosphorylation in the absence of kDNA or presence of azide (Fig 8), but in the absence of ΔΨm, ATP may not reach the cytosol in sufficient quantities to sustain viability: ATP^4-^/ADP^3-^ exchange by the AAC is driven by the concentration gradient of the substrates as well as ΔΨm [73,74]. Resolving which of these scenarios, if any, is correct will require further experimental evidence.

In summary, these experiments demonstrate clear differences in physiology and metabolic capacity of stumpy cells with and without kDNA.

### What defects in mitochondrial function cause the reduced lifespan of stumpy forms lacking kDNA?

Although we found clear evidence for deficiencies in mitochondrial function in kDNA^0^ stumpy cells, correlating any of these deficiencies to the reduced lifespan was not straightforward. The most prominent defect of kDNA^0^ stumpy cells that we identified in this study was lack of a ΔΨm. However, the ability of stumpy cells lacking a ΔΨm (i.e. kDNA^0^ cells or kDNA^+^ cells in the presence of azide) to survive for at least 48 h in medium provided with glucose suggests that sufficient amounts of ATP can be produced via glycolysis in the absence of a ΔΨm, at least in the short term. In the long term, ΔΨm-dependent mitochondrial transport processes such as continued import of the alternative oxidase, are vital for sustained glycolysis in proliferating parasites [7], but this may be less relevant for cell-cycle arrested stumpy forms with their intrinsically limited life span. If loss of ΔΨm does not affect viability of kDNA^+^ stumpy cells, what is the cause of the reduced lifespan in kDNA^0^ stumpy cells? One possibility is an impaired redox balance in the mitochondrial matrix caused by loss of kDNA. At least seven subunits of cI are kDNA-encoded [75], and therefore kDNA^0^ cells will be cI-deficient. Activity of this enzyme is dispensable for slender BSF, at least *in vitro* and in the bloodstream [75], probably in part due to the presence of an alternative type 2 NADH dehydrogenase [76]. Differentiation into stumpy cells has long been known to be associated with a dramatic increase in ‘NAD diaphorase’ activity [9] (an assay for NADH dehydrogenase activity), and we note that both pathways for mitochondrial substrate phosphorylation are dependent on recycling of NADH (Fig 8).

### Conclusions and outlook

Our study shows that kDNA in the sleeping sickness parasite *T*. *brucei* is not required for differentiation into the transmissible stumpy stage, but that it is critical for the longevity of this stage and for generation of its ΔΨm. We identified three important differences to slender BSF *T*. *brucei*: (i) a L262P mutation in the nuclear-encoded ATPase subunit γ does not enable kDNA-independent generation of ΔΨm, most likely because of considerable mitochondrial ATP production; (ii) loss of ΔΨm does not affect the life span of stumpy *T*. *brucei*, presumably because life span is limited by other factors that come into play before loss of ΔΨm-dependent processes can take their toll; and (iii) stumpy form viability depends on kDNA-encoded genes other than F_O_ subunit *a*. Future studies should, for example, assess the consequences of loss of function of respiratory complex I and the F_1_F_O_-ATP synthase on stumpy cell viability with specific genetic mutants and seek to identify the intrinsic factors that limit stumpy cell life span.

## Materials and methods

### Generation of heterozygous γL262P cell lines

Culture-adapted pleomorphic *T*. *brucei* EATRO 1125 AnTat1.1 90:13 parasites [34] were transfected with plasmid pEnT6-γL262P-PURO or pEnT6-γWT-PURO. These plasmids are based on the pEnT6 backbone [77] and contain either F_1_F_O_-ATPase subunit γ (systematic TriTrypDB ID Tb927.10.180) with the L262P mutation (L262Pγ, [16]) or a wild type version (WTγ); they allow the replacement of one endogenous ATPase γ subunit allele in order to generate cell lines containing a single L262Pγ allele (ATPγ/Δatpγ::atpγL262P PURO) or, as a control, WTγ (ATPγ/Δatpγ::atpγWT PURO). The replaced gene is expressed by read-through transcription of the endogenous locus and contains its native 5’ UTR, but the aldolase 3’UTR. For the transfection, the AMAXA Nucleofector II was used with nucleofection solution (90 mM NaH_2_PO_4_, 5 mM KCl, 0.15 M CaCl_2_, 50 mM HEPES, pH 7.3) [78] and program Z-001. *T*. *brucei* EATRO 1125 AnTat1.1 90:13 clones were selected after 4 days and were maintained in 2.5 μg/ml G418, 5 μg/ml hygromycin and 0.1 μg/ml puromycin in HMI-9 medium [79] containing 10% (v/v) fetal calf serum (FCS; Gibco).

The ATPase γ subunit gene was amplified from genomic DNA via PCR, allowing direct Sanger sequencing of the gel-extracted PCR product to confirm the presence or absence of the L262Pγ mutation using primers 5’-CGG CGG CCG CAT GTC AGG TAA ACT TCG TCT TTA CAA AG-3' (forward) and 5'-ATA GGA TCC CTA CTT GGT TAC TGC CCC TTC CCA G-3' (reverse).

### Generation of kDNA^0^ cell lines

WT/L262Pγ cells were treated with 10 nM acriflavine (Sigma) over 3 days; loss of kDNA was assessed by preparing microscope slides and mounting with a cover slip using 50 μl Prolong Gold Antifade with 4’, 6-diamidino-2-phenylindole (DAPI; Life Tech.). To confirm loss of maxicircle genes and of a representative minicircle (type A-like) [16,80] by PCR, total DNA was extracted after expanding the cell culture for a further two days in the absence of acriflavine. The PCR assay was carried out exactly as described in Dean et al. (2013).

WT/L262Pγ kDNA^0^ clone #3 was generated without drug treatment; the cell line lost its kDNA spontaneously after 6 weeks of growth in HMI-9, 10% (v/v) FCS.

### Growth analysis of *T*. *brucei* cell lines *in vitro*

Cells were grown in the presence or absence of 10 nM ethidium bromide (EtBr; Sigma). Cell counts were performed daily using a Beckmann Z2 Coulter counter, and cultures were split to a concentration of 1x10^5^/ml after counting.

### Ethics statement

All animal experiments were carried out in adult MF1 mice after local ethical approval at the University of Edinburgh. All animal experiments were carried out by Caroline Dewar, working under personal license I3997C068 and project licences 60/4373 (Professor Keith Matthews) and 70/8734 (Professor Achim Schnaufer), granted by the UK Home Office under the Animals (Scientific Procedures) Act 1986, section 5.

### Mouse infections with *T*. *brucei*

Sex- and age-matched MF1 mice were infected with *T*. *brucei* EATRO 1125 AnTat1.1 90:13 cells (suspended in 200 μl HMI-9) via intraperitoneal (IP) injection. No immunosuppressant was used. Parasitaemia was monitored by obtaining blood via a tail snip, compressing a drop of blood under a cover slip on a microscope slide, and counting parasites at 400x magnification. Five μl blood was also taken for an immunofluorescence assay and cell cycle analysis. Morphology counts were performed as described [36]. Methanol-fixed blood smear slides were blinded by a colleague with respect to cell line, day and time point to prevent bias. Morphology was scored from these slides independently by two individuals.

Parasitaemia was judged by eye, based on the Rapid Matching method [36,81]. This method entails an upper limit of 64 parasites per field of view, correlating to a density of 2.5x10^8^ cells/ml, above which it becomes difficult to estimate counts accurately.

### Western blotting

Stumpy form trypanosomes were purified from blood using DEAE-cellulose DE52 (Whatman) anionic exchange columns [82] that were preincubated with PSG (44 mM NaCl, 57 mM Na_2_HPO_4_, 3 mM KH_2_PO_4_, 55 mM glucose, pH 7.8) warmed to 37°C. Western blotting and antibody concentrations were as described previously [35]. Anti-EF1α (Millipore) was used at a dilution of 1/7000. Proteins were detected using Enhanced Chemiluminescence reagents (Amersham) and a SRX-101A X-ray developer (Konica Minolta).

### Mathematical model for *T*. *brucei* infection dynamics

A mathematical model described previously [36] was modified to include a parameter for SIF-independent slender to stumpy differentiation.

The model was constructed as follows. Let the concentration of non-committed slender cells at time *t* be *L*(*t*). The initial infection is at time *t* = 0. Non-committed slender cells replicate at rate *α* (i.e., a cell-cycle time of *ln* (2)/*α*)). They are cleared by a time-dependent immune response at rate *r*_*L*_(*t*). They become committed to differentiate at rate *β*_*b*_ + *β*_*f*_*f*(*t*), where *f*(*t*) is SIF concentration, *β*_*b*_ is the background, SIF independent differentiation rate and *β*_*f*_ is the SIF dependent differentiation rate. Therefore, the differential equation that describes the dynamics of non-committed slender forms is:

$$\frac{d}{\mathrm{dt}}L(t)=\left[\alpha -\beta_{b}-\beta_{f}f(t)-r_{L}(t)\right]L(t)$$

Let the age of differentiated cells since becoming committed to differentiation be *a* and let *d*(*a*,*t*) be the age density distribution of differentiated cells at time *t*.

Differentiated cells fall into two classes: i) replicating, committed slender cells, and ii) non-replicating stumpy cells. Committed slender cells replicate at rate *α*, are assumed to be cleared by the immune system at the same rate as non-committed slender cells (*r*_*L*_(*t*)), and develop into stumpy cells at age *τ*_*C*_. Stumpy cells do not replicate, they are assumed to be cleared by the immune response at a different rate *r*_*S*_(*t*), and they die at age *τ*_*S*_. Thus, the partial differential equation that describes the dynamics of the age density distribution of committed cells is

$$\frac{\partial }{\partial t}d(a,t)+\frac{\partial }{\partial a}d(a,t)=-d(a,t)\times \left\{ \begin{array}{cc} r_{L}(t)-\alpha & \mathrm{if}\;0\leq a<\tau_{C}\\ r_{S}(t) & \mathrm{if}\;\tau_{C}\leq a<\tau_{S} \end{array}\right.$$

The boundary conditions on these equations are determined by differentiation of non-committed slender cells into age *a* = 0, i.e., *d*(0,*t*) = [*β*_*b*_ + *β*_*f*_*f*(*t*)]*L*(*t*), and stumpy death at age *τ*_*S*_, i.e., *d*(*τ*_*S*_,*t*) = 0.

Let *C*(*t*) be the total concentration of committed slender cells, let *S*(*t*) be the total concentration of stumpy cells, and let *T*(*t*) be the total concentration of all cells. These are given by:

$$\begin{array}{c} C\left(t\right)=\int d(a,t)\mathrm{da}\\ S\left(t\right)=\int d(a,t)\mathrm{da}\\ T(t)=L(t)+C(t)+S(t) \end{array}$$

SIF is produced by both non-committed and committed slender cells. SIF is removed at rate *γ*. Therefore, the differential equation describing the dynamics of SIF concentration is:

$$\frac{d}{\mathrm{dt}}f(t)=L(t)+C(t)-\gamma f(t)$$

Note that, because SIF is not measured, its concentration is on a dimensionless scale.

The immune response against trypanosomes is multifactorial and highly complex, and only qualitatively understood at best. A detailed mathematical model of the immune response was, therefore, of little use when no data were available to fit to. Instead, we used a simple step function to represent an immune response switching from an inactive to an active state at a time *T* post infection. The strengths of the immune responses against slender and stumpy cells are assumed to be different. They are given by the equations

$$r_{L}(t)=\left\{ \begin{array}{cc} 0 & \mathrm{if}\;t<T\\ \varphi_{L} & \mathrm{if}\;t\geq T \end{array}\right.$$

and

$$r_{S}(t)=\left\{ \begin{array}{cc} 0 & \mathrm{if}\;t<T\\ \varphi_{S} & \mathrm{if}\;t\geq T \end{array}\right.$$

where *ϕ*_*L*_ is the removal rate of slender cells and *ϕ*_*S*_ is the removal rate of stumpy cells.

Naive mice are infected with non-committed slender cells at a concentration *L*_0_. Therefore the initial conditions are *L*(0) = *L*_0_, *d*(*a*,0) = 0 for all *a* and *f*(0) = 0. These imply *C*(0) = *S*(0) = *T*(0) = 0. All variables and parameters are listed in Table 3.

**Table 3:** Variables and parameters used in the mathematical model.

**Independent variables**		
*t*	Time since infection	h
*a*	Age of differentiated cells	h
**Dependent variables**		
*d*(*a*,*t*)	Age distribution of differentiated cells	cells μl^-1^h^-1^
*r*_*L*_(*t*)	Immune-mediated clearance rate of all slender cells	h^-1^
*r*_*S*_(*t*)	Immune-mediated clearance rate of stumpy cells	h^-1^
*f*(*t*)	SIF concentration	dimensionless
*L*(*t*)	Concentration of non-committed slender cells	cells μl^-1^
*C*(*t*)	Concentration of committed slender cells	cells μl^-1^
*S*(*t*)	Concentration of stumpy cells	cells μl^-1^
*T*(*t*)	Total concentration of all cells	cells μl^-1^
**Parameters**		
*α*	Replication rate of slender cells	h^-1^
*β*_*b*_	SIF independent differentiation rate	h^-1^
*β*_*f*_	SIF dependent differentiation rate	h^-1^
*γ*	SIF removal rate	h^-1^
*τ*_*C*_	Lifespan of committed slender cells	h
*τ*_*S*_	Lifespan of stumpy cells	h
*T*	Time until activation of immune response	h
*φ*_*L*_	Immune clearance rate of slender cells	h^-1^
*φ*_*S*_	Immune clearance rate of stumpy cells	h^-1^
*L*_0_	Initial concentration of slender cells	cells μl^-1^

For particular numerical values of the model parameters, the model was solved numerically for each mouse. In order to quantify the fit of the model with these parameters to the data, the log-likelihood of the model solution at each data point by was calculated. Parasite density was estimated by observing a field of cells and estimating the number of parasites in the field. Due to the difficulty of observing many moving parasites in a microscopic field, density estimates were categorised into 0, 1, 2, 3, 4, 5, 6, 8, 10, 12, 16, 20, 24, 32, 48, 64, and 92 parasites per field. Parameter $\rho_{t_{i}}$ describes the expected density of parasites at time *t*_*i*_ (which is obtained from the model). The volume of blood *v*, in a microscopic field is *v* = 25.6 × 10^−8^*μ*l. The expected number of parasites per field therefore is $\lambda =v\rho_{t_{i}}$. The number of parasites *N* in a field is Poisson distributed with parameter *λ*. If *N* equals 0 to 5 then the likelihood of $\rho_{t_{i}}$ is equal to $\frac{\lambda^{N}e^{-\lambda }}{N!}$. If *N* is greater than 5 then it can be assumed that the number of parasites lies within a range. The start of the range is the midpoint between the previous category and the assigned category. For example, if the number of parasites in a field is estimated to be about 48 parasites, then the assigned category is 48, the start of the range is *N*_*l*_ = (32 + 48)/2 = 40 parasites. Similarly, the end of the range is the midpoint between the next category and the assigned category, for example *N*_*u*_ = (48 + 64)/2 = 56. The likelihood of $\rho_{t_{i}}$ is then equal to $\sum_{i=N_{l}}^{N_{h}}\frac{\lambda^{i}e^{-\lambda }}{i!}$ which equals *Q*(*N*_*h*_,*λ*) − *Q*(*N*_*l*_,*λ*) where *Q* is the normalised incomplete Gamma function.

The likelihood function also includes the proportion of parasites that are slender forms at a time *t*_*i*_. The number *X* of parasites that have slender morphology is binomially distributed with parameters *M* and *p*, where *M* is the number of parasites observed and *p* is the predicted proportion that are slender forms (obtained from the model). Thus, the likelihood of $p_{t_{i}}$ is proportional to $p_{t_{i}}^{X_{t_{i}}}{\left(1-p_{t_{i}}\right)}^{M_{t_{i}}-X_{t_{i}}}$.

The parameter posterior distribution was found by multiplying the likelihood, which is the product of likelihoods at each time point, by the prior distributions, which were taken from [36]. The prior on *β*_*b*_ was N_T_(0.01, 0.01^2^), a normal distribution truncated at 0. Samples from the posterior were drawn using an adaptive population based Markov chain Monte Carlo algorithm with power posteriors [83,84].

### Cell cycle analysis

A 5 μl blood sample was pipetted into 100 μl ice cold vPBS (pH 7.4; 137 mM NaCl, 2.7 mM KCl, 10 mM Na_2_HPO_4_, 1.8 mM KH_2_PO_4_, 46 mM sucrose, 10 mM glucose), washed and cells were fixed for 10 min by ice cold 3% (w/v) paraformaldehyde (Fisher). 130 μl 0.2 M glycine was added to allow sample storage. Cells were pelleted, washed in PBS (pH 7.4; 137 mM NaCl, 2.7 mM KCl, 10 mM Na_2_HPO_4_, 1.8 mM KH_2_PO_4_), and resuspended in 500 μl PBS with 100 ng/ml DAPI or 5 μg/ml Hoechst 33342 DNA staining dye (Life Tech.). Cells were analysed by flow cytometry (peak excitation 358 nm, peak emission 461 nm) using a Becton Dickinson LSRII machine with BD FACSDiva software. 2x10^4^ events per sample were measured. Results were analysed with FlowJo software.

### *In vitro* incubation of stumpy forms

Cells were harvested from a mouse infection during peak parasitaemia whilst the population was approximately 90% stumpy form. After purification from blood, parasites were washed in PBS-G (PBS, 6 mM glucose) and resuspended in either HMI-9 containing 10% (v/v) FCS or a modified minimal medium (CMM) [52] containing 10% (v/v) FCS and devoid of glucose. Supplements (25 mM glucose, 25 mM α-KG, 50 mM N-acetyl glucosamine, all from Sigma) were added as required.

### Live/Dead staining

Cells were harvested from culture and washed in sterile warm PBS-G. The pellet was resuspended in PBS-G with 10 μM CFDA-SE (ThermoFisher), and incubated for 15 mins at 37°C. Cells were washed with HMI-9 medium and incubated in HMI-9 for 30 mins at 37°C. Cells were then washed, and fixed with 3.7% (v/v) formaldehyde for 10 min (a detailed paper on validating CFDA-SE staining as a live/dead assay compatible with fixation of cells will be published elsewhere). Fixative was washed out with PBS-G, and cells were resuspended in PBS-G plus 5 μg/ml Hoechst 33342 (Life Tech.). Samples were analysed with excitation peak of 492 nm and emission peak of 517 nm for CFDA-SE on a BD LSRII instrument.

For propidium iodide (PI) staining, 1 μl 500 ng/ml PI was added to the final resuspension before analysis. Samples were analysed at peak excitation at 488 nm and emission at 695 nm.

### Measuring mitochondrial membrane potential (ΔΨm)

The TMRE Mitochondrial Membrane Potential kit (Abcam) was used. All samples were supplemented with 100 nM TMRE and left at 37°C for 20 min. Cells were preincubated with 20 μM carbonyl cyanide-4-(trifluoromethoxy)phenylhydrazone (FCCP) for 10 min or with 0.1–2 mM sodium azide for 2 h. Cells were pelleted, and washed in 0.2% BSA in PBS. Cell pellets were resuspended in 0.2% BSA in PBS containing 5 μg/ml Hoechst 33342 DNA staining dye before analysis on a BD LSRII instrument, with peak excitation at 549 nm and peak emission at 575 nm for TMRE.

### Microscopy

Images were captured using a Retiga 2000R Mono Cooled charged-coupled device camera attached to an Axioscope 2 or Axioimager Z2 (Carl Zeiss MicroImaging, Inc.) using either Plan-Apochromat 63x (1.40 NA) or Plan-Apochromat 100x (1.40 NA) phase-contrast objectives.

## Supporting information

S1 Fig Confirmation of kDNA loss in the WT/L262Pγ kDNA^0^ cell lines, and growth comparison *in vitro*. (A) Where applicable, total DNA was extracted two days after acriflavine treatment. Presence of the following sequences in total cellular DNA was assessed by PCR amplification: maxicircle-encoded genes F_1_F_O_-ATPase subunit 6 (A6), NADH:ubiquinone oxidoreductase subunits 4, 5, and 7 (ND4, ND5, ND7), minicircle type A (MoA), nuclear-encoded gene dihydrolipoyl dehydrogenase (LDH; systematic TriTrypDB ID Tb927.11.16730). (B) Loss of kDNA in acriflavine–treated WT/L262Pγ cells assessed by staining with 4',6-diamidino-2-phenylindole (DAPI) and fluorescence microscopy. (C) Cumulative *in vitro* growth analysis of cells in HMI-9 medium (10% (v/v) FCS) in the absence (sold lines) or presence (dashed lines) of 10 nM ethidium bromide (EtBr); n = 3. Time point zero is defined by the addition of EtBr to treated cells.
(TIF)

S2 Fig A second peak of slender forms arises at day 7 post infection for kDNA^+^, but not for kDNA^0^ cell lines. Blood samples were washed and DAPI stained for cell cycle analysis via flow cytometry. Stumpy forms are cell cycle arrested, and so do not enter G_2_ phase of the cell cycle.
(TIF)

S3 Fig The fit of the mathematical model to the data for infections with *T. brucei* of genotypes WT/WTγ, WT/L262Pγ and WT/L262Pγ (kDNA^0^). The data is represented by filled dots. The coloured lines represent median fits of the model; the shaded regions indicate 95% predictive intervals, where 95% of future data would be predicted to lie according to the model and the data already observed. (A, C, E, G) The mathematical model used involves SIF-dependent and SIF-independent differentiation terms. (B, D, F, H) The mathematical model only includes a SIF-dependent differentiation term.
(TIF)

S4 Fig Fit of the model including only a SIF dependent term for differentiation. (A) Standardised residuals (blue circles) of parasite density and slender fraction, by time, of the model fits with SIF-dependent differentiation only to all mice. Under a true model standardised residuals have an approximately standard normal distribution (i.e., zero mean and unit standard deviation (SD)). Inadequate fit of a model is indicated by its residuals deviating from a standard normal distribution (such as residuals further than ~3 SD from zero, represented by the lightest grey shading, or a set of residuals consistently above or below zero. The red line shows the average, across all mice, of the residuals at a particular time point. (B) Assessment of the quality of fit of the two alternative models to infection data from MacGregor et al., 2011, using the Akaike information criterion (AIC). The AIC measures the quality of a fit of mathematical model to a set of data, taking into account the goodness of fit and the number of parameters estimated in the model. As increasing the number of parameters improves the goodness of fit, AIC penalizes models with more estimated parameters to discourage overfitting. Hence the model with the lowest AIC, i.e. the model with the lowest number of parameters to prevent overfitting, is preferred.
(TIF)

S5 Fig Physiological analysis of cell lines. (A) Cell cycle analysis with Hoechst 33342 dye and flow cytometry to assess slender form (SL) contamination. Stumpy forms (ST) are cell cycle arrested in G_1_ phase. The absence of G_2_ peaks (except in the SL control) suggests that slender contamination was minimal. (B) Establishment of a flow cytometry gate for live/dead staining with PI. 1x10^6^ cells were analysed. Stumpy cells killed by heat treatment (red), live cells (orange) and a mix of live and dead cells (green) were analysed. (C) Measurement of ΔΨm in WT/WTγ stumpy cells maintained *in vitro* in the presence and absence of azide. Cells were incubated in HMI-9 medium for 0, 24 or 48 h, +/- 0.5 mM sodium azide. At each time point, 1x10^6^ cells were stained with TMRE and analysed by flow cytometry. The black line shows the ‘no ΔΨm’ gate which is dictated by the TMRE fluorescence of cells treated with uncoupler FCCP (20 μM; grey population in the background in all panels; note that the grey population is difficult to discern as it almost completely overlaps with the azide-treated populations). The average % cells that retain ΔΨm in the absence of azide treatment is indicated. Left panel: dark green, plus azide; apricot, no azide. Middle panel: magenta, plus azide; yellow, no azide. Right panel: light green, plus azide; purple, no azide. (D) Cells were harvested from mice at maximum parasitaemia, with approximately 90% stumpy forms, and placed in Creek’s minimal medium, supplemented as indicated. GlcNAc, N-acetyl glucosamine. The percentage of live cells after 24 hrs was assessed by PI staining and flow cytometry; n = 3 for each cell line.
(TIF)
