# Supplementary figures and images for: Mitochondrial DNA is critical for longevity and metabolism of transmission stage Trypanosoma brucei
Source: PLoS Pathog. 2018 Jul 18;14(7):e1007195. doi: 10.1371/journal.ppat.1007195 (PMC6066258; doi:10.1371/journal.ppat.1007195)

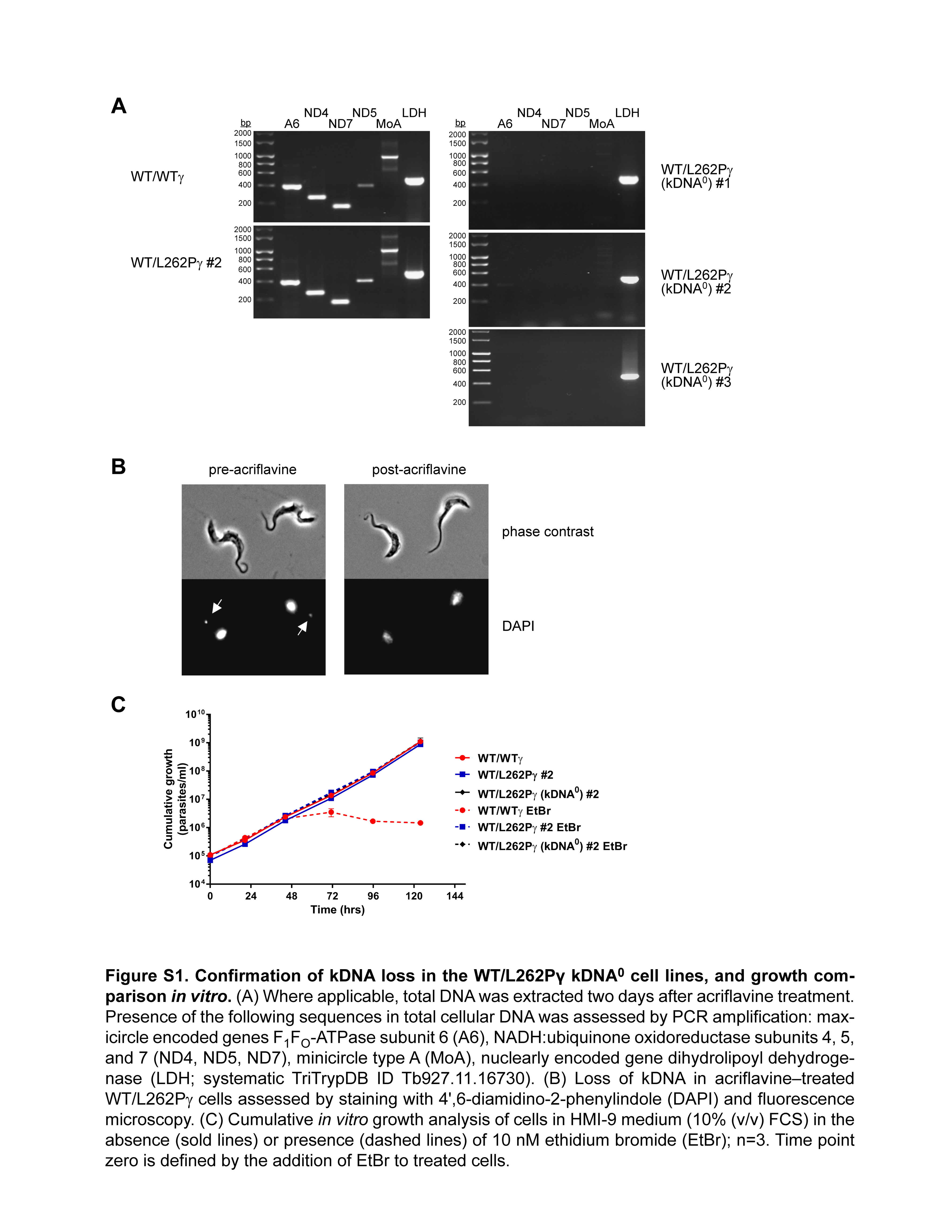

Supplement: S1 Fig — (A) Where applicable, total DNA was extracted two days after acriflavine treatment. Presence of the following sequences in total cellular DNA was assessed by PCR amplification: maxicircle-encoded genes F1FO-ATPase subunit 6 (A6), NADH:ubiquinone oxidoreductase subunits 4, 5, and 7 (ND4, ND5, ND7), minicircle type A (MoA), nuclear-encoded gene dihydrolipoyl dehydrogenase (LDH; systematic TriTrypDB ID Tb927.11.16730). (B) Loss of kDNA in acriflavine–treated WT/L262Pγ cells assessed by staining with 4',6-diamidino-2-phenylindole (DAPI) and fluorescence microscopy. (C) Cumulative in vitro growth analysis of cells in HMI-9 medium (10% (v/v) FCS) in the absence (sold lines) or presence (dashed lines) of 10 nM ethidium bromide (EtBr); n = 3. Time point zero is defined by the addition of EtBr to treated cells. (TIF) [file ppat.1007195.s001.tif]

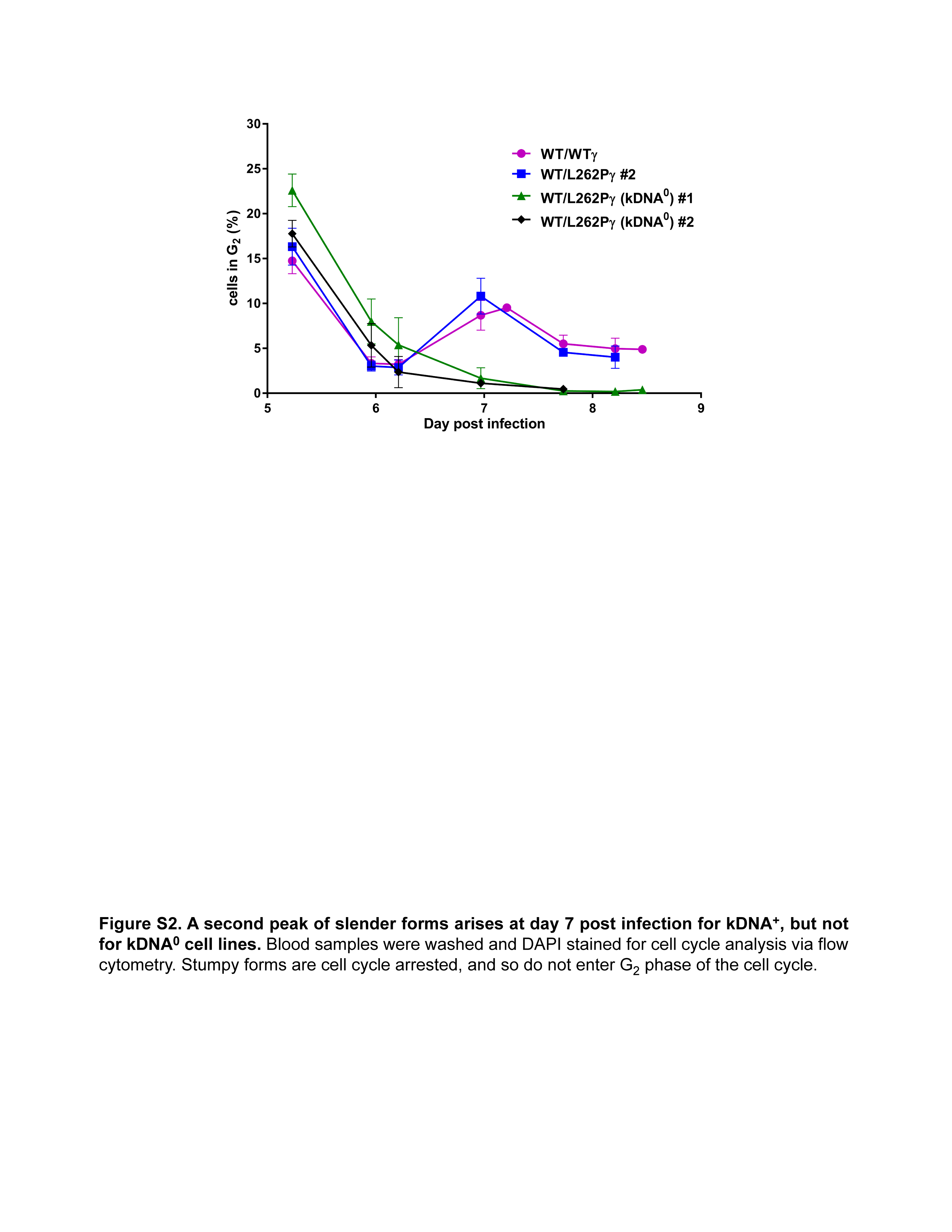

Supplement: S2 Fig — Blood samples were washed and DAPI stained for cell cycle analysis via flow cytometry. Stumpy forms are cell cycle arrested, and so do not enter G2 phase of the cell cycle. (TIF) [file ppat.1007195.s002.tif]

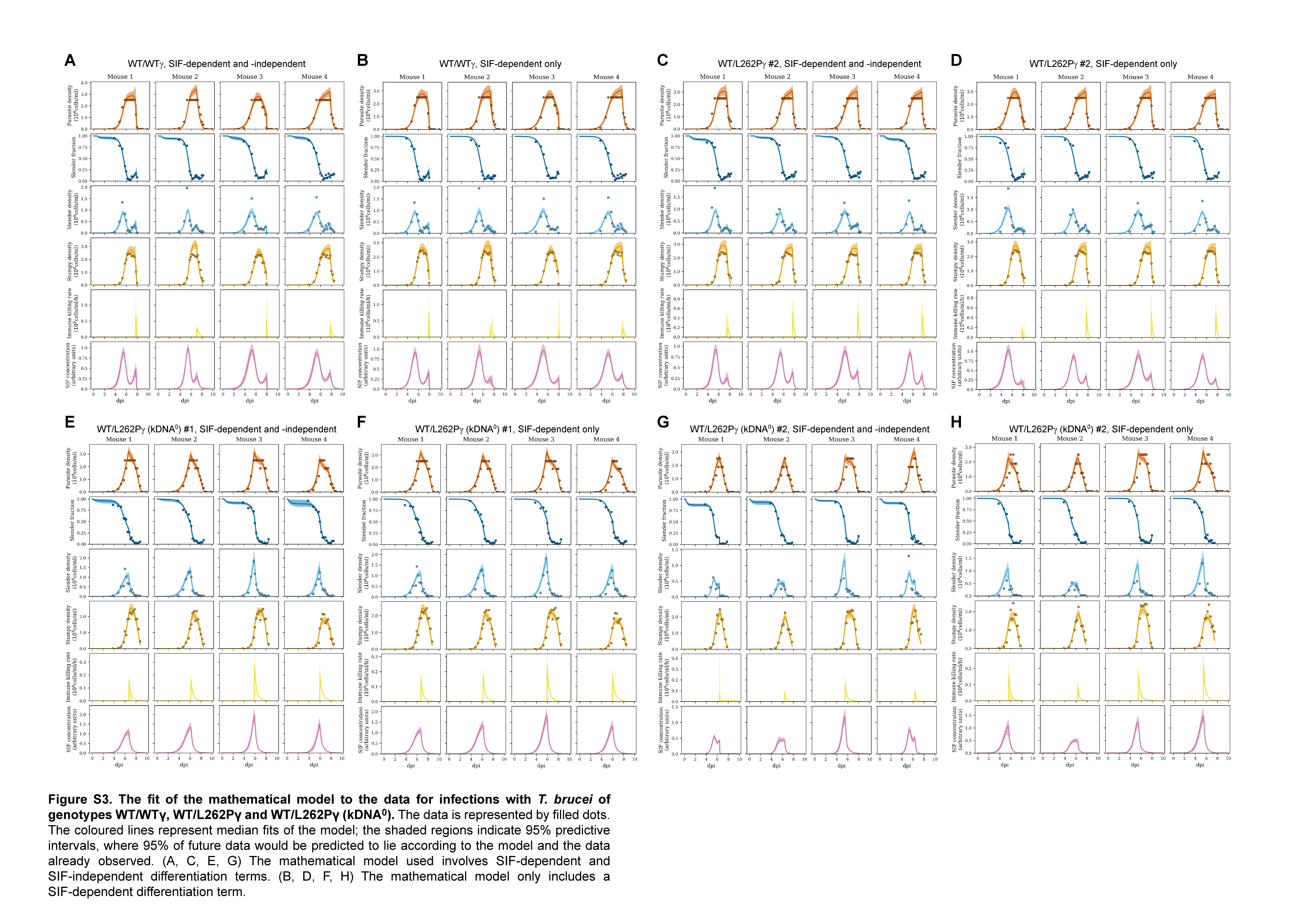

Supplement: S3 Fig — The data is represented by filled dots. The coloured lines represent median fits of the model; the shaded regions indicate 95% predictive intervals, where 95% of future data would be predicted to lie according to the model and the data already observed. (A, C, E, G) The mathematical model used involves SIF-dependent and SIF-independent differentiation terms. (B, D, F, H) The mathematical model only includes a SIF-dependent differentiation term. (TIF) [file ppat.1007195.s003.tif]

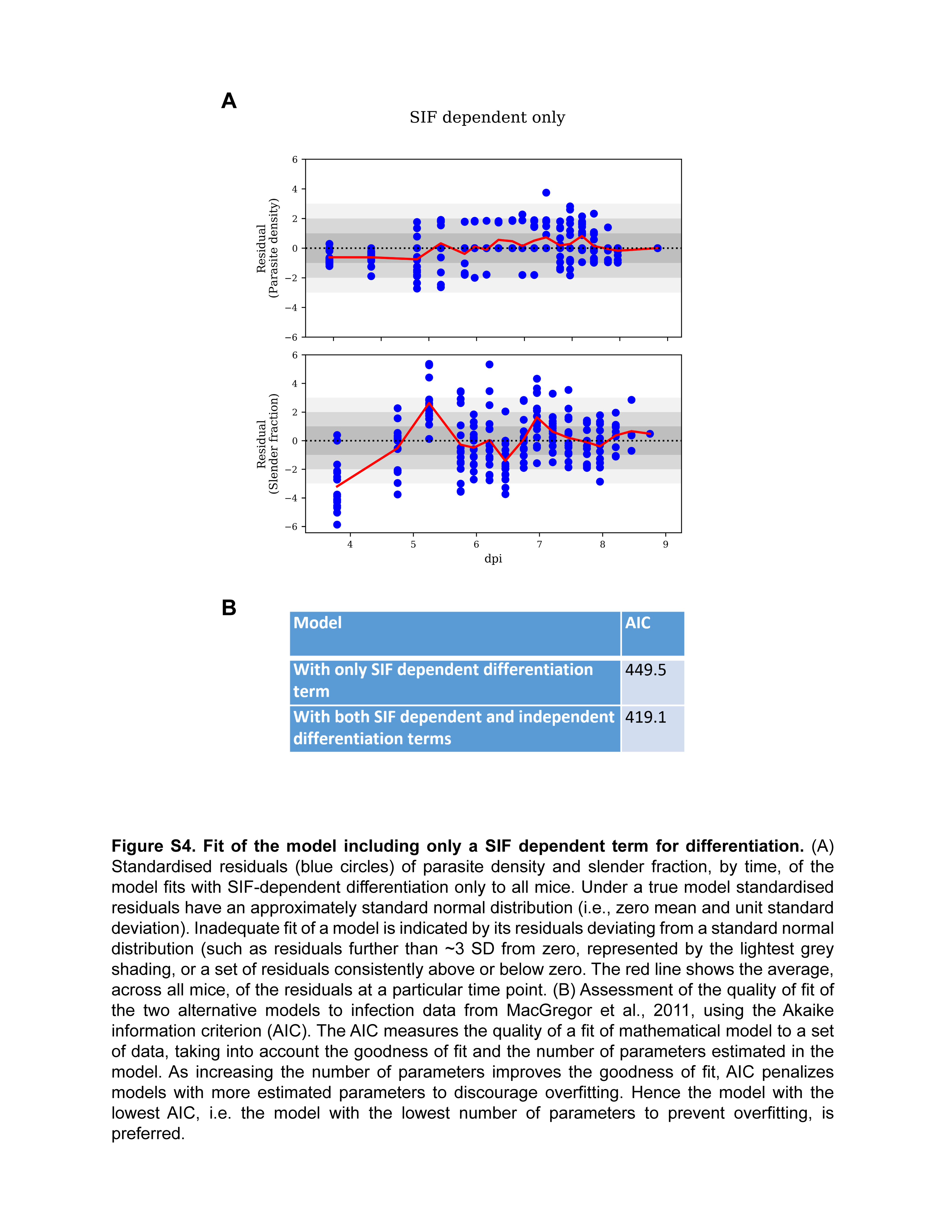

Supplement: S4 Fig — (A) Standardised residuals (blue circles) of parasite density and slender fraction, by time, of the model fits with SIF-dependent differentiation only to all mice. Under a true model standardised residuals have an approximately standard normal distribution (i.e., zero mean and unit standard deviation (SD)). Inadequate fit of a model is indicated by its residuals deviating from a standard normal distribution (such as residuals further than ~3 SD from zero, represented by the lightest grey shading, or a set of residuals consistently above or below zero. The red line shows the average, across all mice, of the residuals at a particular time point. (B) Assessment of the quality of fit of the two alternative models to infection data from MacGregor et al., 2011, using the Akaike information criterion (AIC). The AIC measures the quality of a fit of mathematical model to a set of data, taking into account the goodness of fit and the number of parameters estimated in the model. As increasing the number of parameters improves the goodness of fit, AIC penalizes models with more estimated parameters to discourage overfitting. Hence the model with the lowest AIC, i.e. the model with the lowest number of parameters to prevent overfitting, is preferred. (TIF) [file ppat.1007195.s004.tif]

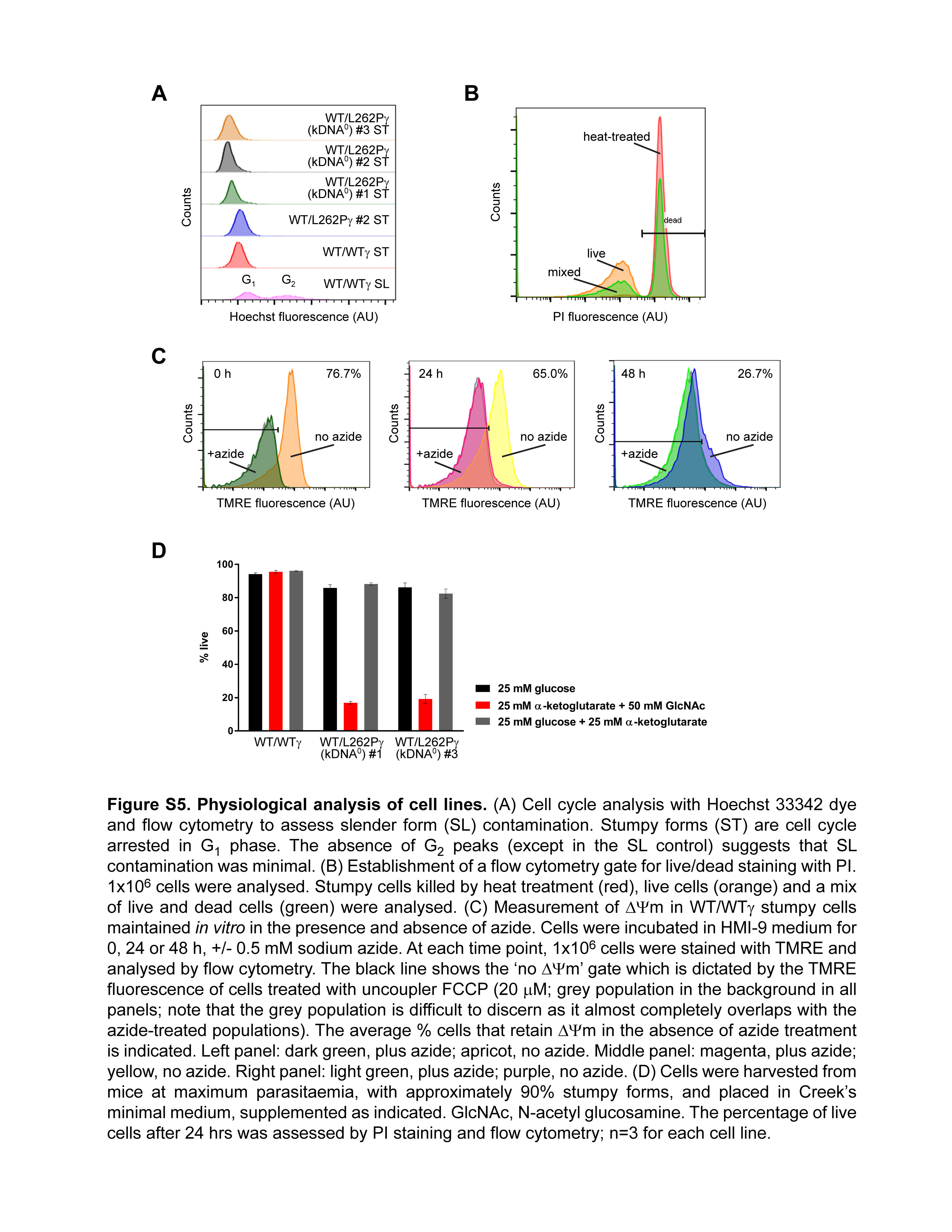

Supplement: S5 Fig — (A) Cell cycle analysis with Hoechst 33342 dye and flow cytometry to assess slender form (SL) contamination. Stumpy forms (ST) are cell cycle arrested in G1 phase. The absence of G2 peaks (except in the SL control) suggests that slender contamination was minimal. (B) Establishment of a flow cytometry gate for live/dead staining with PI. 1x10^6^ cells were analysed. Stumpy cells killed by heat treatment (red), live cells (orange) and a mix of live and dead cells (green) were analysed. (C) Measurement of ΔΨm in WT/WTγ stumpy cells maintained in vitro in the presence and absence of azide. Cells were incubated in HMI-9 medium for 0, 24 or 48 h, +/- 0.5 mM sodium azide. At each time point, 1x10^6^ cells were stained with TMRE and analysed by flow cytometry. The black line shows the ‘no ΔΨm’ gate which is dictated by the TMRE fluorescence of cells treated with uncoupler FCCP (20 μM; grey population in the background in all panels; note that the grey population is difficult to discern as it almost completely overlaps with the azide-treated populations). The average % cells that retain ΔΨm in the absence of azide treatment is indicated. Left panel: dark green, plus azide; apricot, no azide. Middle panel: magenta, plus azide; yellow, no azide. Right panel: light green, plus azide; purple, no azide. (D) Cells were harvested from mice at maximum parasitaemia, with approximately 90% stumpy forms, and placed in Creek’s minimal medium, supplemented as indicated. GlcNAc, N-acetyl glucosamine. The percentage of live cells after 24 hrs was assessed by PI staining and flow cytometry; n = 3 for each cell line. (TIF) [file ppat.1007195.s005.tif]
